# Characterizing molecular and behavioral changes arising from ROMK potassium channel deficiency in the cerebellum

**DOI:** 10.3389/fnbeh.2025.1681149

**Published:** 2026-01-26

**Authors:** Jacopo Agrimi, Aidan J. Dunphy, Justin C. Zhong, Wenxi Zhang, Naeem Sbaiti, Daniel R. Turner, Lucia Bernardele, D. Brian Foster, Brian O’Rourke, Nazareno Paolocci, Kyriakos N. Papanicolaou

**Affiliations:** 1 Division of Cardiology, Department of Medicine, The Johns Hopkins University School of Medicine, Baltimore, MD, United States; 2 Department of Biomedical Sciences, University of Padova, Padua, Italy

**Keywords:** anxiety-like behavior, Bartter’s syndrome, cerebellum, GFAP, granule cells, KCNJ1, motor coordination, potassium channels

## Abstract

The cerebellum is critically involved in both motor coordination and affective regulation, and growing evidence suggests that cerebellar dysfunction contributes to neuropsychiatric disorders. While much attention has focused on synaptic signaling and calcium homeostasis, the role of potassium channels in cerebellar function remains relatively understudied. Here, we investigated the role of the potassium channel ROMK (renal outer medulla K^+^ channel) in cerebellar signaling and behavior using cre/loxP gene knockout in Pcp2cre-expressing cells. Surprisingly, ROMK expression was detected in a distinct cell population within the cerebellar granule layer, rather than in Purkinje cells, yet this expression was effectively targeted by Pcp2cre-mediated recombination. Mutant mice showed normal Purkinje cell density and soma size, but increased dendrite diameter. At the molecular level, we observed downregulation of cerebellar subtype-specific genes and potassium channel subunits, along with changes in markers of translational signaling. Increased presence of GFAP-positive cells further suggested underlying neuronal stress in the ROMK-deficient cerebellum. Behaviorally, ROMK-deficient mice exhibited clear impairments in motor coordination and heightened anxiety-like behavior in the elevated plus maze (EPM). Our findings link ROMK loss to molecular and cellular remodeling in the cerebellum and support the idea that ROMK contributes to neural circuits that regulate complex behaviors, providing a framework for further studies in this direction.

## Introduction

K^+^ channels are vital for neuronal excitability because they help establish the resting membrane potential, shape action potentials, and restore the membrane potential after neuronal firing. For example, the voltage-gated K^+^ channels (Kv) Kcnc1 and Kcnc3 are implicated in motor coordination by regulating membrane excitability in cerebellar neurons (Hurlock et al., 2008; Hurlock et al., 2009; Cook et al., 2021). Conversely, the inward rectifier K^+^ channels (Kir) are gated by various physiological factors such as Mg^2+^, polyamines, ATP, or interaction with binding proteins (Hibino et al., 2010).

Background Kir channels (predominantly Kir2.x) control neuronal electrical excitability and glial cell K^+^ flux (Neusch et al., 2003), but much less is known about the specific role of Kir1.x channels in the brain. One member of the family, Kir1.1, also known as Kcnj1 or ROMK, is predominantly expressed in the kidney, where it participates in ion exchange and K^+^ secretion (Welling and Ho, 2009). Loss-of-function mutations in ROMK cause Bartter syndrome (type II), a neonatal renal condition characterized by prematurity, hypokalemia, deficient sodium re-absorption, hypotension and metabolic alkalosis (Cunha and Heilberg, 2018). Early in the description of the syndrome, it was reported that Bartter patients may exhibit neuronal complications including motor and cognitive dysfunction (Simopoulos, 1979). In addition to the kidney, ROMK expression is also detected in the CNS (Shuck et al., 1994; Kondo et al., 1996), including the cortex, the hippocampus and the brainstem (Kenna et al., 1994; Wu et al., 2004; Yamamoto et al., 2008; Shcherbatyy et al., 2015).

Despite ROMK’s well-characterized roles in the kidney, its potential roles in the CNS remain unclear. Stable overexpression of ROMK negatively impacts action potential firing in hippocampal neurons and decreases the resting membrane potential, suggesting that the channel dampens neuronal excitability (Nadeau et al., 2000). Furthermore, phosphorylation and activation of ROMK can mediate the effects of the anti-epileptic drug levetiracetam (Lee et al., 2008). Additionally, alterations in ROMK expression have been observed in certain psychiatric conditions. For example, the expression of ROMK is upregulated in the striatum of patients with major depression (Smolin et al., 2012). Furthermore, a genome-wide association study found significant evidence for an association between the region harboring the ROMK gene locus (11q24) and the personality trait of openness (Amin et al., 2013). Finally, in some rare cases of Bartter syndrome, patients experience psychiatric symptoms including auditory and visual hallucinations, anxiety, and mania (Özdemir et al., 2014; Ridout et al., 2016).

The cerebellum is well-known to regulate motor coordination and balance (Manto et al., 2012). However, evidence shows that the cerebellum has roles in other behavioral domains, including stress response and associative learning (Sacchetti et al., 2002; Utz et al., 2015; Ernst et al., 2019; Frontera et al., 2020; Bohne et al., 2022). Purkinje cells (PCs) are a distinct and highly specialized class of GABAergic inhibitory neurons that serve as the sole output of the cerebellar cortex to the deep cerebellar nuclei, where they integrate and fine-tune circuit activity. In addition to their roles in motor coordination, recent studies demonstrate that modulating PC activity can influence affective behaviors, including aggression and anxiety (Jackman et al., 2020). PCs receive excitatory input from the parallel fibers of cerebellar granule cells, which represent the most abundant neuronal population in the brain. Granule cell activity is shaped primarily by excitatory inputs from mossy fibers and modulated by inhibitory feedback inputs from Golgi cells, reflecting the structural and functional complexity of neuronal circuits within the cerebellum (Sillitoe and Joyner, 2007). This interconnected granule cell–Purkinje cell axis offers a useful framework for exploring how potassium channel activity might affect behavioral function.

In a recent study, Okada and coworkers performed central administration of tertiapin-RQ and observed enhanced anxiety-like behavior and altered motor activity in mice (Okada et al., 2020). The same study demonstrated that tertiapin-RQ had a higher specificity for inhibiting ROMK, rather than Kir2.1, suggesting that ROMK inhibition influences different behavioral responses (Okada et al., 2020). However, simulation experiments show that tertiapin-RQ is also a highly potent Kir2.1 inhibitor (Hilder and Chung, 2013), so it could in theory affect the resting membrane potential of all neurons. Furthermore, another tertiapin-Q variant (tertiapin-LQ) known to block ROMK (Ramu et al., 2008), is shown to affect cerebellar Purkinje neuron functions (Johansson and Hesslow, 2020), although these effects were ascribed to targeting Kir3.1. Hence, there is still uncertainty concerning the specificity of the blockers, and the cell types within the brain that are responsible for the behavioral responses remain elusive.

Our previous work established both germline and tissue-specific models of ROMK deficiency, including a whole-body knockout that recapitulated features of neonatal Bartter syndrome, and a cardiomyocyte-specific model (Papanicolaou et al., 2020). Building on these tools, we sought to explore the role of ROMK in the nervous system, focusing on its potential contribution to behavior. To this end we developed a conditional knockout model targeting ROMK in cerebellar Pcp2cre-expressing cells and sought to determine whether loss of ROMK alters animal behavior and to explore the underlying molecular changes associated with such dysfunction.

## Materials and methods

### Mouse generation and genotyping

The generation of mice with the core exon of ROMK flanked by loxP sites (ROMK^flox^) was previously described (Papanicolaou et al., 2020). These mice were crossed with Pcp2cre^Tg^ mice (also referred to as L7Cre-2) expressing cre in Purkinje neurons [Jackson Laboratory stock 004146 (Barski et al., 2000; Sługocka et al., 2017)]. The resulting ROMK^flox/flox^; Pcp2cre^Tg^ mice were crossed with the reporter mouse strain encoding a tandem dimer (td) of the fluorescent protein Tomato knocked-into the Rosa26 locus (tdTom^KI^, also known as Ai9, Jackson Laboratory stock 007909). The expression of the knocked-in transgene is suppressed due to the presence of a Lox-STOP-Lox cassette, that is excised only in cre-expressing cells, serving as a reporter of cell-specific cre expression and activation.

For mouse genotyping, crude genomic DNA was extracted from tail clips using DirectPCR (Cat. No. 102-T, Viagen). PCR was performed with OneTaq DNA polymerase mastermix (NEB, Cat. No. M0486) in the presence of 0.2 μM gene-specific primers and diluted genomic DNA (200-fold final dilution). PCR products were resolved on ethidium-bromide agarose gels and imaged with UV. The primer sequences, loci genotyped and PCR size of their expected products are shown in Supplementary Table 1.

Behavioral assays were performed with male mice to avoid sex-related variability in locomotor and anxiety measures for this initial study. Histology, immunofluorescence, and molecular assays (qPCR, immunoblots) used mixed-sex cohorts that included both males and females being represented in each assay. Accordingly, these datasets are not sufficiently powered for sex-stratified analyses.

### RNA extraction, cDNA synthesis, and real-time quantitative PCR

RNA was extracted from cerebellar tissue using Trizol according to manufacturer’s protocol with modifications to add extra washing steps with ethanol. Extracted RNA was diluted down to a concentration of 160 ng/μl. cDNA was synthesized using Luna RT SuperMix Kit according to the manufacturer’s instructions with an initial input of 800 nanograms of RNA per reaction mixture. Reactions with no reverse-transcriptase (NoRT) were performed in parallel, in order to control for potential genomic DNA carryover. Resulting cDNA samples were diluted 40-fold to be used for real-time quantitative PCR. Technical duplicates were done for each sample where 2 microliters of cDNA were combined with LUNA dye (Cat. No. M3004S,) and 500 nM forward and reverse primers. Real-time quantitative PCR was performed using BioRad CFX384 Touch Real-Time PCR Detection System using the following amplification protocol: 45 cycles of 15 s at 95 °C (denaturation) followed by 30 s at 60 °C (elongation). Evaluation of melt curves was performed at the end of the runs to detect the quality of amplification and potential formation of primer dimers. Resulting Ct values for each sample/gene were expressed relative to B2M as the reference gene to calculate ΔCt values. All primer sequences are shown in Supplementary Table 2.

For the mouse oxidative stress PCR array (Cat No. PAMM-065ZE, Qiagen), 1,280 ng of whole cerebellum RNA was reverse transcribed into cDNA according to manufacturer specifications, including a genomic DNA elimination step with buffer GE. PCR plates (384 wells) containing gene-specific primers were loaded with 10 microliters of mastermix containing RT2 SYBR Green and cDNA from each individual sample. Real-time quantitative PCR was conducted using a BioRad CFX384 Touch Real-Time PCR Detection System using the following amplification protocol: 10 min at 95 °C (initial denaturation) followed by 40 cycles of 15 s at 95 °C (denaturation) and 1 min at 60 °C (elongation). Raw Ct values from each well were assigned to the appropriate gene using an online tool provided by the manufacturer. Resulting Ct values from each well were expressed relative to B2M to obtain normalized Ct values. Further statistical analysis on the expression of the 88 genes was performed by log2-transformation of the normalized values and then performing the Linear Models for Microarray data (limma) in R including empirical Bayesian statistics (eBayes) and the Benjamini-Hochberg adjustment of the *p*-values (Ritchie et al., 2015; Phipson et al., 2016).

### Histological analysis

Freshly dissected brain and kidney tissues were examined for the expression of the tdtomato reporter using a fluorescent stereomicroscope (Leica). In addition, thinly sliced cerebellum and kidneys were imaged with a confocal fluorescence microscope (Olympus FV300RS) using the 2 × objective lens (Universal Plan Apochromator, 0.08 Numerical Aperture, 6.2 mm working distance) or the 10 × objective lens (Universal Plan Apochromator, 0.4 Numerical Aperture, 3.1 mm working distance). In other experiments, the mouse cerebellum was prepared for histological analysis using a fixative solution consisting of 4% paraformaldehyde (PFA) in PBS, which was perfused at a constant flow through a cardiac puncture. Following fixation, the cerebellum was embedded in OCT compound and frozen sections were obtained with a cryotome. Nissl (cresyl violet) staining was applied to indicate the different cerebellar cell populations. Stained sections were automatically scanned and digitized at the 10 × magnification (Proscia).

### Immunofluorescent staining, confocal imaging and analysis

Animals were deeply anesthetized with 3,3,3 tribromoethanol (avertin) at a dose of 250 mg/kg administered via intraperitoneal injection. Once unresponsive to toe pinch, mice were transcardially perfused using a syringe pump at a flow rate of 10 mL/min with ice-cold phosphate-buffered saline (PBS), followed by 4% paraformaldehyde (PFA) in PBS. Brains were carefully extracted and post-fixed by immersion in 4% PFA overnight at 4 °C. Following post-fixation, brains were transferred to a 30% sucrose solution in PBS for cryoprotection and stored at 4 °C until they sank to the bottom of the container, typically 24–48 h. The tissue was then embedded in Optimal Cutting Temperature (OCT) compound, frozen on dry ice, and stored at −80 °C until sectioning. Coronal, or sagittal sections (50 μm thick) were obtained using a Tanner TN60 cryostat maintained at −20 °C. Sections were serially collected and stored until further processing.

For standard antibody staining, sections were first blocked/permeabilized for 1 h at room temperature in a solution containing 10% normal goat serum (NGS, Cell Signaling Technology, catalog number: 5425) and 0.05% Triton X-100 in PBS. Following this step, sections were incubated with primary antibodies diluted in 5% NGS in 0.05% Tween-20 PBS (PBS-T) overnight at 4 °C with gentle orbital shaking. For a detailed description of antibodies used in this study please see Supplementary Table 3. For immunofluorescence, the primary antibodies used were anti-calbindin (1:1000, Proteintech, Cat. No. 66394-1-Ig, Host: Mouse), anti-calbindin (1:1000, Proteintech, Cat. No. 14479-1-AP, Host: Rabbit), anti-GFAP (1:250, Cell Signaling Technology, Cat. No. 3670, Host: Mouse) and anti-rpS6 (1:500, Cell Signaling Technology, Cat. No. 2217, Host: Rabbit). Following primary antibody incubation, sections were washed with PBS-T (4 × 20 min with orbital shaking) to remove unbound antibodies and then incubated with species-specific secondary antibodies conjugated to Alexa fluorophores, diluted in antibody blocking solution. Incubations with secondary antibodies were performed at room temperature with gentle orbital shaking for 1 h, protected from light. This was followed by staining with Hoechst 33342 (2 μg/mL stock solution, Thermo Fisher Scientific, Cat. No. H3570) for 10 min in the dark. Sections were then washed in PBS-T (4 × 20 min with orbital shaking) and mounted on glass slides using Vibrance anti-fading medium (Vector Laboratories).

For ROMK staining in the cerebellum, hippocampus, cortex, and brainstem, free-floating sections were rinsed in PBS and blocked for 1 h at room temperature in 10% NGS, 0.05% Triton X-100 in PBS with gentle orbital shaking. Following washing of the permeabilization solution in PBS-T, the sections were placed in primary antibody solution containing anti-ROMK (Proteintech, Cat. No. 20953-1-AP) diluted 1:250 in 5% NGS in PBS-T and incubated overnight at 4 °C with gentle shaking. After washing the primary antibody with PBS-T (4 × 20 min with orbital shaking), sections were incubated for 2 h at room temperature with a secondary poly-HRP-conjugated anti-rabbit antibody. Tyramide signal amplification was performed using Thermo’s Alexa Fluor 488 SuperBoost Kit (Cat. No. B40922), according to the manufacturer’s instructions. After 7.5 min of amplification, reactions were quenched with stop solution and sections washed 4 × 20 min in PBS-T with orbital shaking and protecting from light. The sections were next incubated with anti-Calbindin (1:1,000; Proteintech, Cat. No. 66394-1-Ig, host: mouse) in 5% NGS/PBS-T overnight at 4 °C with gentle shaking, washed in PBS-T (4 × 20 min) and incubated for 2 h with secondary Alexa Fluor 568-conjugated goat anti-mouse (1:1000). After PBS-T washes (4 × 20 min), nuclei were counterstained with Hoechst 33342 as above, and sections were mounted on glass slides for confocal imaging.

Images were acquired using an Olympus FV3000 confocal microscope equipped with 405 nm, 488 nm, 561 nm, and 640 nm lasers. To achieve high spatial resolution and three-dimensional reconstruction by maximum z-stack projection, thick sections were scanned at optimal intervals to cover the entire thickness of the sections (Botta et al., 2022). For image acquisition we used the 20x objective (Universal Plan Super Apochromator, 0.75 numerical aperture, 0.6 mm working distance, 360 nm Z step), or the 40 × silicon oil immersion objective (Universal Plan Super Apochromator, 1.25 numerical aperture, 0.3 mm working distance, 240 nm, Z step).

For ROMK quantification in the cerebellum, we systematically imaged lobules IV-VIII of the cerebellar vermis at 20 × on a Fluoview FV3000, centering each field of view on the same anatomical landmark in Control and PKO sections. Z-stacks were acquired from non-overlapping 20 × confocal fields (636.4 × 636.4 μm), maximum-projected in Fiji, and puncta clusters (>5 puncta) within the granule cell layer were manually counted (sections lacking clusters were scored as zero). This approach ensured consistent regional sampling across all specimens.

To quantify Purkinje cell (PC) anatomical features, we acquired non-overlapping 40 × confocal fields (318.2 × 318.2 μm) from anatomically matched regions within lobules IV–VIII of the cerebellar vermis in both Control and PKO brains, ensuring equal sampling across genotypes. Z-stacks were converted to maximum projections in Fiji/ImageJ. PC density was determined by counting the number of calbindin-stained cells with a clearly visible soma and proximal primary dendrite within a given field of view, then dividing by the width of the FOV. PC soma size was calculated using the wand/tracing tool and the Analyze>Measure functions of Fiji. The diameter of the primary dendrites was measured across multiple locations of clearly visible primary dendrites using the ‘straight line’ function of Fiji.

To quantify GFAP signal, 20 × confocal images (636.4 × 636.4 μm) were acquired from sagittal sections of Control and PKO cerebella. Multiple fields were collected per section, guided by the presence of GFAP-positive cells and restricted to consistent regions adjacent to the granule cell layer, primarily within the white matter of lobules IV–VIII. These fields were selected to ensure comparable anatomical positioning across genotypes. Images were analyzed as single optical planes and thresholded in Fiji/ImageJ using the Yen algorithm to isolate GFAP-positive signal. Following thresholding, the GFAP-positive signal was quantified using the Analyze>Measure>Area and Integrated density functions of Fiji.

For ROMK quantification in the kidney, transverse sections were used to capture anatomically matched regions of the renal cortex and outer stripe of the outer medulla in both Control and PKO samples. Images were acquired at 40 × magnification (FOV: 318.2 × 318.2 μm) from consistently positioned fields within these regions. Images were thresholded using the Yen algorithm in Fiji/ImageJ, and the ROMK-positive area and integrated density were quantified using the Analyze>Measure>Area and Integrated density functions. All quantitative analyses were performed blind to genotype.

### Behavioral tests

Behavioral experiments were performed in the Johns Hopkins School of Medicine behavioral core according to previous studies (Agrimi et al., 2019; Agrimi et al., 2023). Mice were housed in rooms with inverted light/dark cycle (12/12 h), temperature (21 ± 0·5 °C) and relative humidity (55% ± 2%) and had free access to food and water. Mice were allowed to adjust for 30–60 min before each experiment in a dark behavioral room. Experiments were performed blinded to the genotype of the animals.

### Triple horizontal bars test

The test apparatus consists of three parallel bars placed horizontally at different heights, allowing the animal to explore and traverse them. The mouse is placed on the bars, and its ability to maintain balance and coordination while navigating across the bars is assessed. The test is scored based on the time taken by the animal to traverse the bars, the number of foot slips or falls, and the overall stability observed (Deacon, 2013).

### Rotarod test

Mice were placed on a rotarod (Rotamex, Columbus Instruments) with a starting speed of 4 rpm and an accelerating rate of 6 rpm/min. The time required for a mouse to lose balance and fall from the rotarod was recorded as latency to fall. Daily tests were performed over a period of 5 days and each mouse was given three trials per day with a 2-min interval between each trial (Deacon, 2013).

### Elevated plus maze

The cross-shaped maze consists of four arms; two of which are protected by barriers on the sides (closed arms, 30 cm × 5 cm × 15 cm) and two arms that are exposed (open arms, 30 cm × 5 cm). The maze is elevated 40 cm above the floor. This test assesses the rodent’s conflict between the instinct to hide in the closed arms of the maze (aversion to open spaces) and the instinct to venture in the open arms of the maze (explore new environments). The tests started by placing the mouse in the central area and letting it freely explore the maze for 5 min. Experiments were recorded by a camera placed above the apparatus. Analysis of the behavioral parameters was performed by ANY-maze software.

### Western blot analysis of mouse cerebellum

The cerebellar tissue was thoroughly homogenized using a polytron in RIPA buffer (comprising 150 mM NaCl, 1.0% IGEPAL CA-630, 0.5% sodium deoxycholate, 0.1% SDS, and 50 mM Tris, pH 8.0, Sigma, Cat. No. R0278). The homogenization buffer was supplemented with 1 mM PMSF, along with 1 × protease and phosphatase inhibitors (Millipore, Sigma; cOmplete, mini, EDTA-free, and PhosSTOP, respectively). The homogenates were then centrifuged at 4 °C to separate the soluble fraction. The protein concentration in the supernatant was quantified using the bicinchoninic acid assay (Pierce, Cat. No. 23225).

For the subsequent Western Blot analysis, samples were prepared by mixing with 50 mM DTT and 1 × LDS sample buffer (Thermo Fisher Scientific, NP0008). Each lane was loaded with 20 μg of cerebellar protein and run on Nu-PAGE Bis-Tris 4 to 12% gradient gels (Thermo Fisher Scientific, Cat. No. WG1403). Following electrophoresis, proteins were transferred onto nitrocellulose membranes (Thermo Fisher Scientific, iBlot2 transfer stacks, Cat. No. IB23001). The membranes were stained using REVERT 700 Total Protein Stain (LI-COR, Cat. No. 926-11010) to visualize the total protein loading and blocked in 5% bovine serum albumin (BSA) in TBS for 1 h at room temperature.

Primary antibodies (diluted 1,000:1 in 5% BSA and 0.1% Tween 20-TBS) were incubated with membranes overnight at 4 °C. Post-incubation, the membranes were thoroughly washed with TBS-T. Subsequently, IR Dye-conjugated host-specific secondary antibodies were applied (dilution of 5,000:1 in 5% BSA and 0.1% Tween20-TBS). The specific antibodies used in this study are detailed in Supplementary Table 3. Detection and quantification of band intensities were accomplished using the Odyssey system and Image Studio Lite software (LI-COR). Normalization for protein loading was achieved by comparing the band intensities to the total protein loading.

Urine samples were collected from sex- and age-matched Control and PKO mice by spontaneous urination. Preparation of samples for urinary protein analysis was performed in western blot sample buffer as previously described (Suzuki et al., 2021) and blots were quantified for total protein content using the REVERT 700 total protein reagent and digital scanning as described above.

## Results

### ROMK expression in cerebellum and gene-targeting strategy to generate mice with neuronal deficiency of ROMK

**Figure 1:**
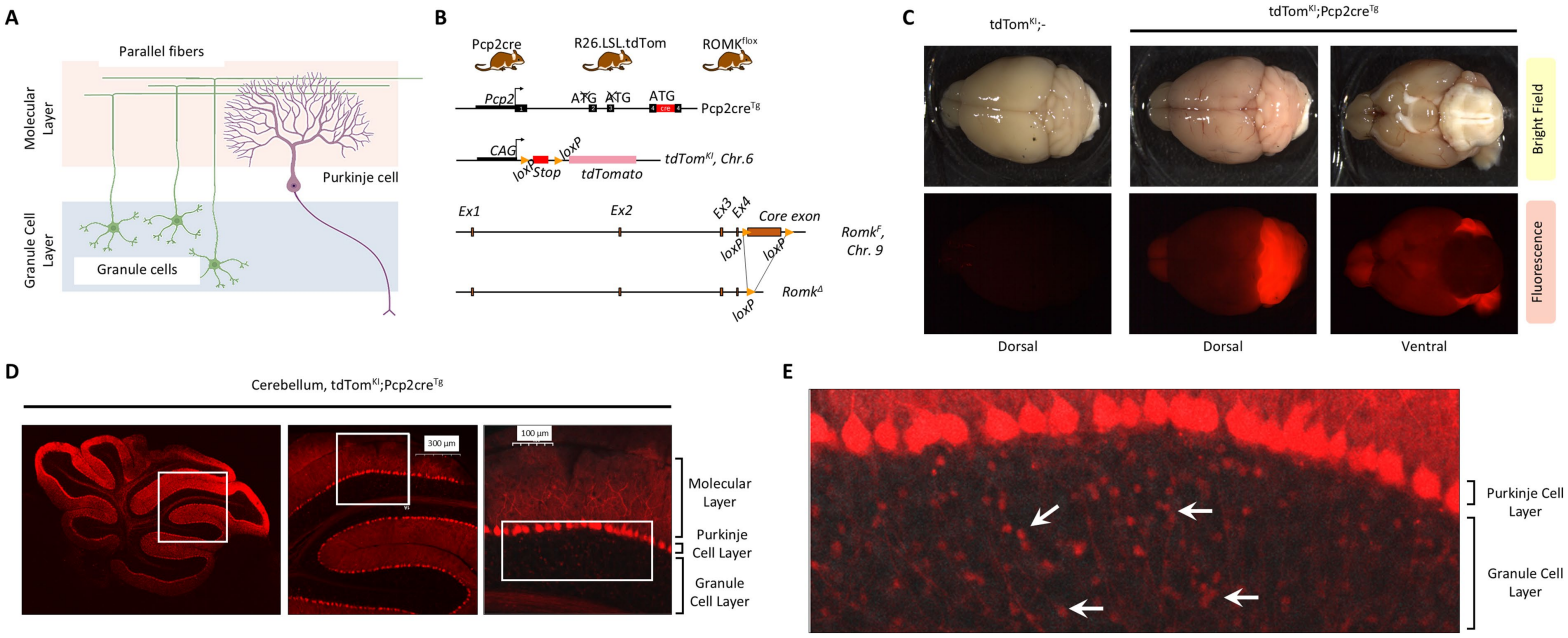
Genetic approach for conditional deletion of ROMK in the mouse cerebellum. **(A)** Simplified organization of the cerebellum to indicate the three major layers (molecular layer, Purkinje cell layer and the granule cell layer) and the functional relationship between granule cells and Purkinje cells (source: BioRender). **(B)** Schematic of the gene deletion strategy to generate conditional ROMK knockout mice in Pcp2cre-expressing cells that also enable the expression of the cell-tracing fluorescent reporter tdTomato. CAG; synthetic promoter containing the cytomegalovirus early enhancer, the chicken *β*-actin promoter and the splice acceptor of the rabbit beta-globin gene. LSL; lox-stop-lox cassette placed upstream the tdTomato reading frame. **(C)** Dorsal and ventral views of freshly dissected whole brain in tdTomato^KI^; − and tdTomato^KI^; Pcp2cre^Tg^ mice showing fluorescence predominantly in the cerebellum. Images were acquired using a fluorescent stereomicroscope. **(D)** Mid-sagittal view of the whole cerebellum of tdTomato^KI^; Pcp2cre^Tg^ mouse showing prominent tdTomato expression in the Purkinje cell layer and the molecular layer. **(E)** Zoomed-in view highlighting the Purkinje cell layer and the adjoining granule cell layer. Distinct puncta (white arrows) within the granule layer appear to express tdTomato, indicating CRE activity in that layer as well. Images were acquired using an FV3000 Olympus confocal microscope using the 4x objective.

The cerebellar cortex is organized into three distinct layers—molecular, Purkinje cell, and granule cell layers—each comprising specialized neuronal populations that contribute to cerebellar signal processing (Figure 1A). Granule cells, located in the innermost layer, give rise to parallel fibers that ascend to the molecular layer, where they form excitatory synapses with the dendritic arbors of PCs, the principal output neurons of the cerebellar cortex (Figure 1A). Molecular layer interneurons such as basket and stellate cells modulate PC activity, while Golgi cells within the granule layer regulate granule cell input, together orchestrating the finely tuned balance of excitation and inhibition necessary for cerebellar function. To investigate the role of ROMK in the cerebellum, we utilized conditional ROMK^flox/flox^ mice (Papanicolaou et al., 2020). These mice were bred with Pcp2cre^Tg^ mice (also referred to as L7Cre-2), a transgenic line driving the expression of Cre recombinase in PCs, retinal bipolar cells, and other neurons (Barski et al., 2000; Witter et al., 2016; Sługocka et al., 2017; Jahncke and Wright, 2024). To monitor the cell type specificity of Cre expression in this model, we introduced via breeding a knock-in allele expressing the fluorescent protein tdTomato in the Rosa26 locus (tdTom^KI^, also known as Ai9, Jackson Laboratory stock 007909) (Madisen et al., 2010). A schematic of the gene targeting strategy is shown on Figure 1B.

To establish the spatial pattern of Pcp2cre activity in this model, we examined brains of tdTom^KI^; Pcp2cre^Tg^ mice. As shown in Figure 1C, macroscopic fluorescence imaging indicated strong activation of tdTomato fluorescence in the mouse cerebellum, with lower expression in the olfactory bulbs and diffuse, or no expression, in other brain regions. Higher magnification imaging confirmed the strong expression of tdTomato in PC cell bodies and dendritic projections (Figure 1D). Interestingly, however, we also observed tdTomato expression in cells of the granule layer (Figure 1E, arrows). It is therefore likely that in our model, Pcp2cre is expressed not only in PCs, but also in other cerebellar neurons of the granule layer.

**Figure 2:**
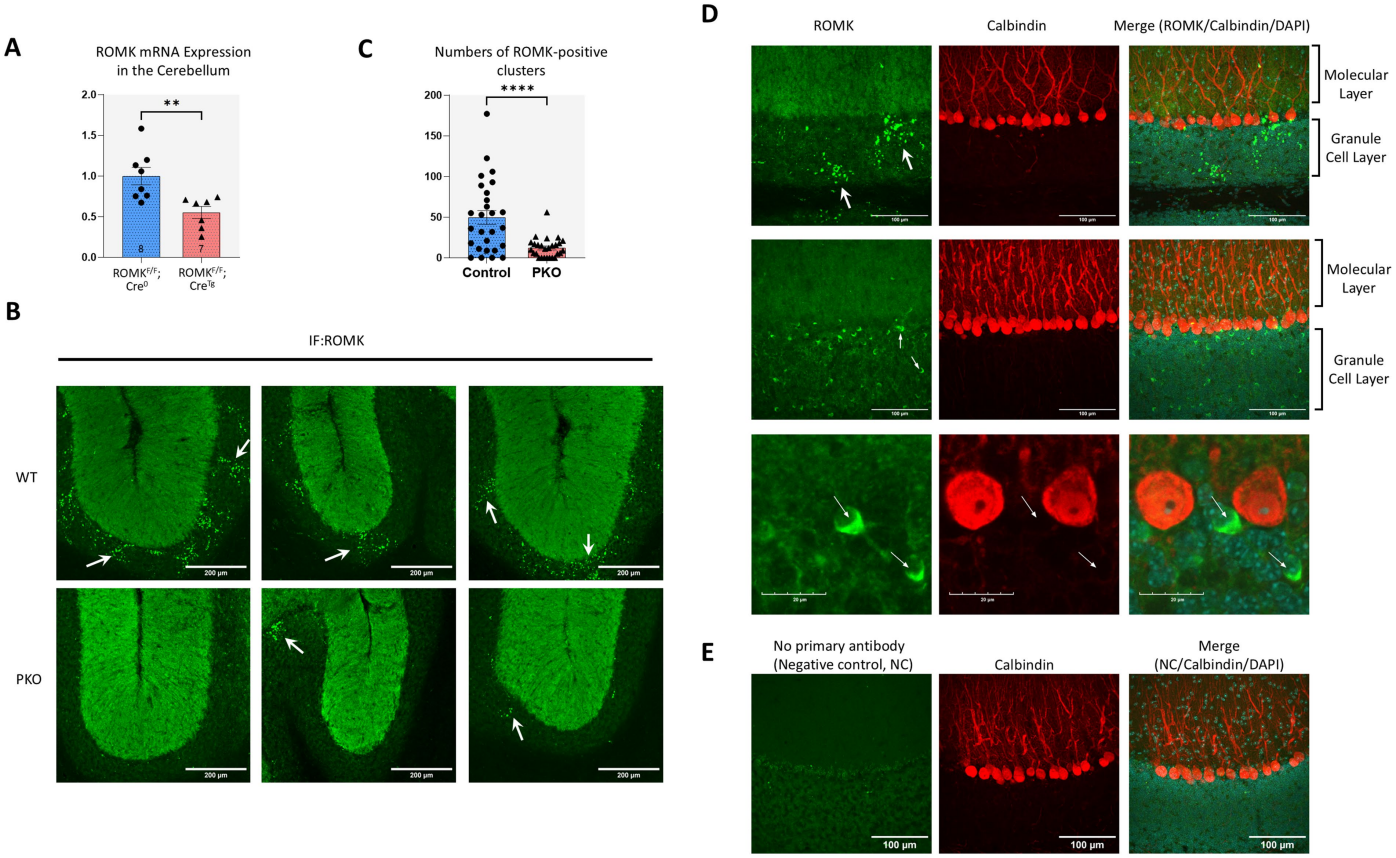
Cerebellar expression of ROMK and its conditional loss in ROMK^F/F^; Pcp2cre^Tg^ mice. **(A)** Quantitative real-time PCR analysis of cerebellar extracts in control and PKO mice (*n* = 8 and *n* = 7 replicates, respectively). Expression of ROMK was normalized to the housekeeping gene Beta-2 Microglobulin (B2M). Comparisons were performed with a two-tailed *t*-test, **p < 0.01. **(B)** Representative images of cerebellar sections immunostained for ROMK following tyramide signal amplification. Arrows indicate ROMK-expressing puncta clustered within discrete regions of the granule cell layer. Signal was most prominent in the granule layer, with no or minimal puncta appearing in the molecular layer (evident by autofluorescence). Images were acquired blindly from non-overlapping regions of the cerebellar cortex with the 20 × objective. Scale bar, 200 μm. **(C)** Quantification of ROMK-positive puncta in control and PKO cerebellum. Puncta were counted only if they appeared in clusters of more than five distinct ROMK-immunoreactive spots within the granule cell layer; regions lacking clusters were scored as zero. The graph shows the number of clustered ROMK puncta across multiple regions obtained from the cerebellum of three mice per genotype, analyzed blindly. ****p < 0.0001, by two-tailed *t*-test. **(D)** Higher magnification view of cerebellar sections dually labeled for ROMK and Calbindin. Maximum intensity z-stack projections were created using a 40 × objective to visualize ROMK (green) and Calbindin (red), serving as Purkinje cell marker. ROMK-positive clusters or ROMK-expressing cells are evident within the granule cell layer (white arrows). Calbindin-positive Purkinje cells show no detectable ROMK signal. **(E)** Negative control (NC). Cerebellar sections were processed without the ROMK primary antibody but otherwise identically (HRP-conjugated secondary, Alexa Fluor 488 tyramide) and imaged with identical acquisition settings (magnification, laser power) as in **(D)**. Calbindin staining was performed unchanged. Scale bar: 100 μm.

Next, we examined the extent of ROMK depletion in the cerebellum of ROMK^flox/flox^; tdTom^KI^, Pcp2cre^Tg^ and their cre-negative littermates (ROMK^flox/flox^; tdTom^KI^;-). Using quantitative real-time PCR, we found a marked reduction of ROMK mRNA (~45% lower ROMK expression, *p* = 0.0049, in cre-expressing cerebellum, Figure 2A). These data demonstrate that the gene-targeting strategy used here effectively reduces ROMK expression in the cerebellum. Mice with the genotype ROMK^flox/flox^; tdTom^KI^; Pcp2cre^Tg^ –engineered to delete ROMK in Pcp2cre-expressing cells—are hereafter referred to as PKO and their cre-negative littermates as Control. In addition to assessing for ROMK mRNA levels, we also utilized immunofluorescent staining to assess ROMK protein expression in the cerebellum of these mice. This approach revealed punctate ROMK-expressing regions within the granule layer that were abundant in Control cerebellum but nearly absent in PKO cerebellum (Figure 2B). The frequency of these punctate ROMK-positive regions was significantly lower in PKO (~75% lower, *p* < 0.0001, Figure 2C). Furthermore, closer inspection of the immunofluorescence images showed that ROMK reactivity is observed in cells within the granule cell layer, consistent with expression in one or more cell populations located in this region, but not in PCs (Figure 2D). To evaluate staining specificity, we performed negative control staining reactions without the ROMK primary antibody but otherwise identically to experimental sections. When imaged in parallel with identical acquisition settings, these controls showed only background-level signal, supporting the specificity of the ROMK staining pattern (Figure 2E). Taken together, these findings indicate that a population of cells within the cerebellar granule layer endogenously expresses ROMK, and that Pcp2Cre activity in this region significantly reduces ROMK signal in PKO.

To evaluate regional specificity of the Pcp2cre-targeted ROMK deletion, we examined ROMK immunolabeling in hippocampus and cortex regions of Control and PKO brains (Supplementary Figure 1A). Examining ROMK expression in the hippocampus did not reveal significant signal, and this was the case in both Control and PKO brains (Supplementary Figure 1B, hippocampus). By contrast, in areas of the cortex we could detect discrete ROMK-expressing cells, and the appearance and abundance of these cells was similar between Control and PKO brains (Supplementary Figure 1C, cortex). Further examinations of the sagittal brain sections revealed regions of the brainstem that again contained discrete ROMK-expressing cells that were equally present in Control and PKO brains (Supplementary Figure 1D, brainstem). These findings are also consistent with previous work documenting ROMK presence in the medulla oblongata (Schultz et al., 2003; Wu et al., 2004), including nerve cell bodies and glial cells of chemosensory areas (Yamamoto et al., 2008). Taken together, these results identify extra-cerebellar areas of the brain with discrete ROMK expression; however, the frequency of ROMK-positive cells, their overall appearance and signal intensity are similar between Control and PKO, supporting the view that there is no off-target ROMK ablation outside the cerebellum in this model of Pcp2cre-mediated gene targeting.

### PKO cerebellum exhibits normal overall structure with mild underlying abnormalities in PC dendrite diameter

**Figure 3:**
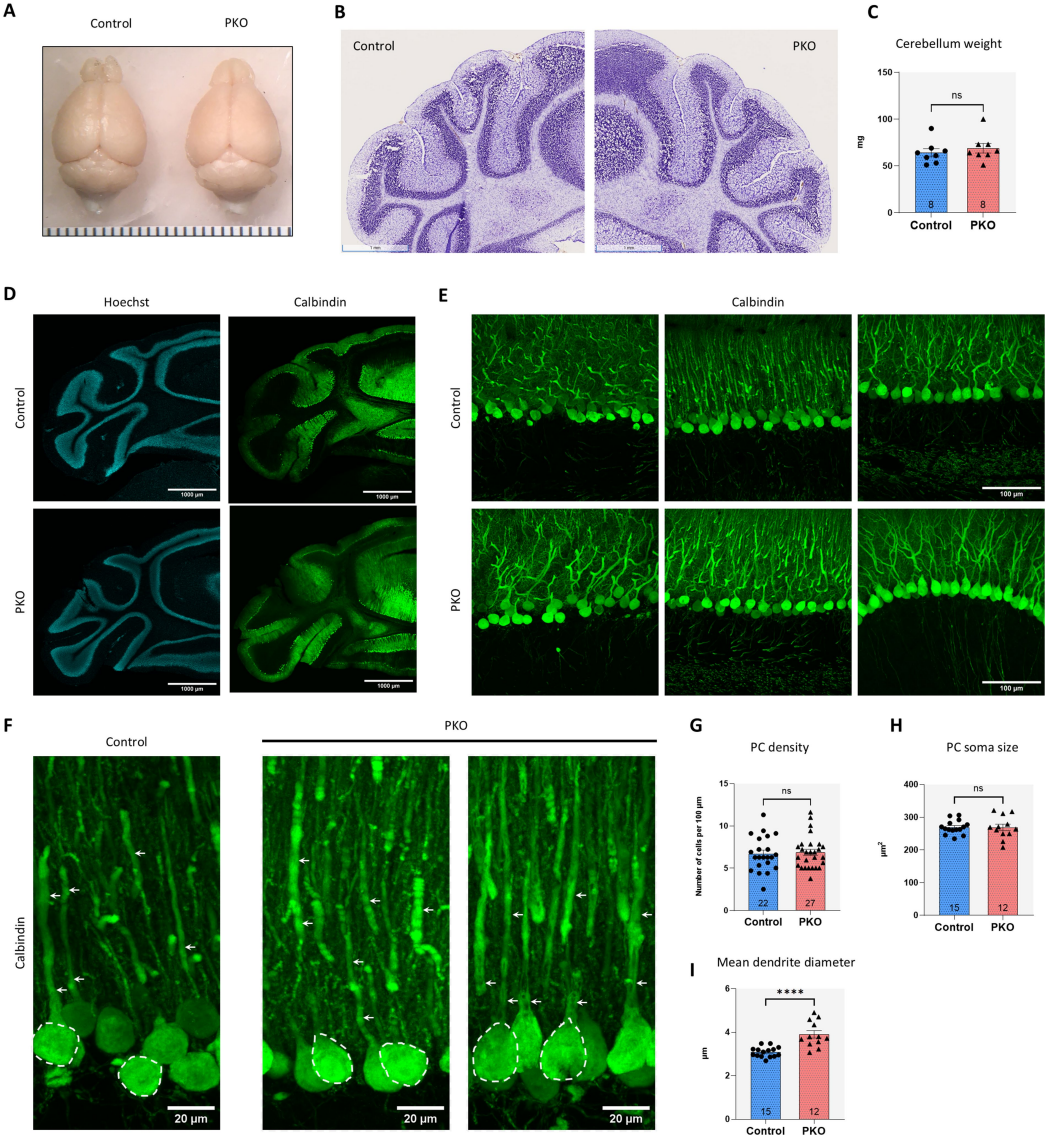
Overall preservation of cerebellar architecture with subtle PC dendritic abnormalities in PKO mice. **(A)** Overall brain structure appears grossly normal with no overt abnormalities in brain size, shape, or regional organization, indicating preserved global neuroanatomy in PKO mice. **(B)** Nissl-stained coronal sections in control and PKO cerebellum at the mid-level, showing the structures of vermis and lobules. Scale bar: 1 mm. **(C)** Cerebella from control and PKO mice were isolated and measured for their overall mass. The study cohort consisted of 8 control and 8 age-matched PKO mice. **(D)** Low magnification (×2) confocal images of control and PKO cerebellum coronal sections stained with the PC marker calbindin and counterstained with Hoechst. Views reveal typical foliation and laminar organization in both control and PKO cerebella. The scale bar is 1,000 μm. **(E)** PCs and their dendrites were visualized in detail by staining cerebellar sections for calbindin. Sections were imaged with the 40 × objective through multiple confocal planes, and the resulting z-stacks were combined into maximum projection images to capture the full extent of PC morphology. Scale bar: 100 μm. **(F)** Zoomed regions showing the morphology of PCs in control and PKO cerebellum. The PC soma is outlined in selected cells with a broken line and the arrows indicate the dendrites. **(G–I)** Quantitative analysis of calbindin-stained cerebellar sections from control and PKO mice assessed for PC density, PC soma size, and PC dendrite diameter. Analyses were conducted using Fiji on at least three sections per brain from two independent animals per genotype. Bar graphs represent aggregated data from multiple fields of view per genotype, as indicated. Statistical comparisons were performed with unpaired two-tailed *t*-test. Ns: not significant, ****p < 0.0001.

Previously, we found that mice with global deficiency of ROMK exhibited reduced brain mass (Papanicolaou et al., 2020), raising the possibility that ROMK plays a role in brain development and growth. Therefore, we asked whether the Pcp2cre-mediated loss of ROMK in our model led to any gross abnormalities in the brain and cerebellum. However, examination of gross appearance showed that PKO brains are indistinguishable from Control brains (Figure 3A). Nissl staining of cerebellar sections further revealed no overt structural differences between the groups, and cerebellar mass was comparable between Control and PKO mice (Figures 3B,C).

Extensive work has shown that mature Purkinje cell dendritic arborization critically depends on signals from granule cells (van der Heijden and Sillitoe, 2021). To gain deeper insights into potential circuit abnormalities in the PKO cerebellum, we performed confocal imaging of cerebellum sections labeled with the PC marker calbindin (Figure 3D). Higher magnification images obtained from different regions of the cerebellum were used to assess PC density (i.e., the number of PCs present over a given distance within the cerebellum cortex, Figure 3E). However, this analysis did not reveal any significant changes in PC density between the two genotypes (Figure 3G). Furthermore, we analyzed calbindin images for the parameters of PC soma size and PC dendrite diameter (Figure 3F). This analysis showed that the PC soma size was similar between Control and PKO (Figure 3H), however the mean dendrite diameter was significantly higher in the PKO group (3.09 vs. 3.90 μm, *p* < 0.001, Control vs. PKO, Figure 3I). Taken together, these results show that PCs are not undergoing loss or overt degeneration in PKO cerebellum. However, the observed increase in dendrite diameter could be a compensatory trophic response to alterations within the cerebellum circuit, potentially reflecting dysfunction in upstream support from granule cells or other cerebellar cell types.

The BDNF/TrkB signaling axis is critical for cerebellar neuron growth and maturation (Minichiello and Klein, 1996; Schwartz et al., 1997), whereby BDNF, secreted by cerebellar granule cells, acts on PCs to regulate their arborization and dendritic spine density (Morrison and Mason, 1998; Carter et al., 2002; Bosman et al., 2006). We therefore examined BDNF and TrkB protein abundance in cerebellum extracts from Control and PKO mice. However, we did not find any significant changes in these two signaling mediators (Supplementary Figures 2A–C). Consistently, no changes were apparent for BDNF mRNA abundance in the PKO cerebellum (Supplementary Figure 2D). Furthermore, we examined by western blotting the status of the signaling mediator Erk and the transcription factor Creb, known to be important in cerebellum development and neuroplasticity (Thomas and Huganir, 2004; Samuels et al., 2009; Sanchez-Ortiz et al., 2014), including roles in dendrite growth and development (Hamad et al., 2023). However, analysis of the phosphorylation status of Erk and Creb did not reveal significant changes in PKO cerebellum (Supplementary Figure 2E). Given ROMK’s potential role in mitochondrial function (Foster et al., 2012; Laskowski et al., 2019; Papanicolaou et al., 2020), we examined protein subunits of the mitochondrial electron transport chain and the ATP synthase as potential indicators of underlying mitochondrial dysfunction. This investigation, however, did not reveal significant differences between Control and PKO cerebellum (Supplementary Figure 2F). Lastly, we examined the autophagy markers Ulk1, p62/Sqstm and LC3B, which were not altered in the PKO cerebellum (Supplementary Figure 2G).

The ribosomal protein S6 (rpS6) is a key component of the 40S ribosomal subunit involved in protein synthesis, it is a downstream effector of the mTOR signaling pathway, and is often used as a neural activity and plasticity marker (Jaworski and Sheng, 2006). Furthermore, reduced activity of rpS6 in the cerebellum is associated with defects in PC dendrite development and morphology (Liu et al., 2020). Examination of confocal images fluorescently labelled for the PC marker calbindin and the rpS6, confirmed the widespread expression of rpS6 in Purkinje cells, but also showed that rpS6 was expressed in some cells of the granule cell layer (Supplementary Figures 3A,B). Interestingly, assessment by western blot identified a moderate but significant increase in rpS6 abundance in PKO cerebellum (Supplementary Figures 3C,D).

### PKO cerebellum exhibits decreased expression of neuronal markers and K^+^ channel genes

To gain further insight into the molecular changes underlying the PKO cerebellum, we examined the expression of various gene targets using quantitative real time PCR (Table 1). First, we examined a panel of neuronally enriched genes, expressed in PCs, granule cells, unipolar brush cells, Golgi cells, and others (Kozareva et al., 2021). Accordingly, we found a significant reduction in the expression of several of these markers (e.g., Dner, Gria1, Grid2, Homer3, Pcp2, Slc1a6, Table 1), suggesting broad circuit-level alterations within the cerebellar cortex that may involve PCs, granule cells, and other neuronal populations.

**Table 1:** Expression of selected genes in PKO cerebellum.

Gene name	Alternate gene name	*p*-value	Significant?	Fold change (PKO/control)	Direction of regulation	Expression in cerebellar subtypes
Neuronal-specific genes						
***Calb1***		0.0317	✓	0.6191	↓	PC
***Calb2***		0.0444	✓	0.6607	↓	Gr, UBC
***Dner***		0.0046	✓	0.6801	↓	PC, MLI, UBC, G,
***Gria1***	*GluR1*	0.0022	✓	0.7019	↓	PC, B
***Grid2***	*GluD2*	0.0287	✓	0.5971	↓	PC, Gr, B, AC
***Homer3***	*Vesl-3*	0.0231	✓	0.5859	↓	PC
***Pcp2***	*L7*	0.0461	✓	0.5930	↓	PC
***Slc1a6***	*EAAT4*	0.0062	✓	0.6026	↓	PC
Potassium channels						
*Kcna4*	*Kv1.4*	0.3431		1.4792		PC
*Kcna5*	*Kv1.5*	0.2721		1.6880		E
*Kcnb1*	*Kv2.1*	0.4749		1.5315		ND
***Kcnc1***	*Kv3.1*	0.0459	✓	0.6126	↓	Gr, MLI, PLI
*Kcnc3*	*Kv3.3*	0.0697		0.7143		PC
*Kcnd1*	*Kv4.1*	0.7298		0.9569		ND
***Kcnd2***	*Kv4.2*	0.0052	✓	0.6417	↓	G, Gr, PLI, UBC, OPC
***Kcnd3***	*Kv4.3*	0.0012	✓	0.6597	↓	PC, Gr, MLI
*Kcnh2*	*HERG*	0.2648		0.4571		G, UBC
*Kcnj2*	*Kir2.1*	0.3100		1.2739		UBC
*Kcnj3*	*Girk1*	0.3591		0.8617		A, B, Gr, UBC
*Kcnj5*	*Girk4*	0.3599		0.8072		G
*Kcnj6*	*Girk2*	0.6114		0.9267		G, UBC
*Kcnj9*	*Girk3*	0.0918		0.7915		ND
***Kcnj12***	*Kir2.2*	0.0273	✓	0.6627	↓	Gr
***Kcnq1***	*Kv7.1*	0.0264	✓	0.6771	↓	G, MLI
*Kcnq3*	*Kv7.3*	0.5649		0.6824		A, MLI, ODC, OPC
Other channels and channel regulators						
***Asic1***		0.0313	✓	0.7865	↓	MLI
*Kcnip2*		0.3979		2.4753		UBC
*Rgs6*		0.3888		1.1345		A, E, G, PLI, UBC
*Scn1a*	*Nav1.1*	0.2003		0.8610		G, MLI, PLI, PC
Stress-related transcription factors						
*Atf4*		0.8404		1.0843		
*Creb1*		0.0779		0.8017		
*Xbp*		0.5120		0.8583		
*Xbp/s*		0.9106		0.9637		
Mitochondrial genes						
*Atp5o*		0.6460		0.9537		
*Cox5b*		0.7554		1.0389		
*Ndufb5*		0.3214		0.8972		
*Trap1*		0.1265		0.8656		
*Ccdc51*		0.0644		0.8260		
*Drp1*		0.3946		0.8969		
*Fis1*		0.5866		0.9405		
***Mfn1***		0.0199	✓	0.7536	↓	
*Mfn2*		0.1273		0.8982		
*Opa1*		0.3411		0.9046		
*Afg3l2*		0.1478		0.8902		
*Hspd1*		0.3356		0.9330		
*Hspe1*		0.9894		0.9983		
mTOR pathway						
*Eif4e*		0.3166		0.8875		
***Ptpn11***		0.0059	✓	0.7658	↓	
*Rictor*		0.1117		0.7762		
*Rps6ka3*		0.2096		0.8251		
***Rptor***		0.0017	✓	0.7232	↓	
***Tsc1***		0.0259	✓	0.8298	↓	
***Tsc2***		0.0049	✓	0.8054	↓	

Genes exhibiting fold regulation (PKO vs. Control) with a *p*-value < 0.05 are shown in bold. *n* = 8 control and *n* = 7 PKO. Cerebellar subtypes: PC, Purkinje cells; Gr., granule cells; A, Astrocytes; B, Bergmann cells; E, endothelial cells; G, Golgi cells; Gr, Granule cells; ND, not detected in the reference study; PLI, Purkinje layer interneurons; MLI, molecular layer interneurons; ODC, oligodendrocyte; OPC, oligodendrocyte progenitor cells; UBC, unipolar brush cells. Nomenclature of cerebellar subtypes was obtained from Kozareva et al. (2021). A threshold of average expression of >1 was used to define cell specific expression.

We also reasoned that lack of ROMK in the PKO cerebellum could potentially trigger changes in the expression of other potassium channel genes (Kcn) as a compensatory mechanism to restore ion homeostasis. We therefore examined the expression of various Kcn genes. While a uniform and generalized change in the expression of these genes was not detected, a select group of Kcn genes exhibited lower expression in PKO cerebellum, including Kcnc1, Kcnd2, Kcnd3, Kcnj12 and Kcnq1 (Table 1). Relevant to these findings, a progressive downregulation of Kcn genes is observed in mouse models of spinocerebellar ataxia (Dell’Orco et al., 2017). It is noted that some of these genes are expressed across diverse cerebellar cell populations including PCs, granule cells, Purkinje layer interneurons, unipolar brush cells, Golgi cells, and others (Table 1).

Previously, it was demonstrated that ROMK deficiency in the kidney is associated with altered activity of the channel Kcnma1 (Calcium-Activated K^+^ Channel, also known as BK or maxi K) (Bailey et al., 2006). Moreover, deficiency of Kcnma1 in mice leads to gait defects and reduced rotarod performance (Meredith et al., 2004), and Kcnma1 mutations in humans are associated with neurobehavioral abnormalities (Park et al., 2022), including cerebellar defects (Tabarki et al., 2016; Du et al., 2020). Considering these findings, we assessed the expression of Kcnma1 and found a mild, yet significant, reduction in Kcnma1 protein abundance in PKO cerebellum (Supplementary Figures 4A,B). On the other hand, the ABC transporter Cystic Fibrosis Transmembrane Conductance Regulator (Cftr), whose function is also associated with that of ROMK (McNicholas et al., 1996), did not change significantly in PKO cerebellum (Supplementary Figure 4C).

Defects in mitochondrial metabolic activity, mitochondrial dynamics and mitochondrial protein turnover have been associated with neuronal abnormalities and cerebellar dysfunction (Jahani-Asl et al., 2007; Quintana et al., 2010; Kageyama et al., 2012; Ripolone et al., 2018; Motori et al., 2020; Rumyantseva et al., 2022). Moreover, a ROMK isoform may be expressed in the inner mitochondrial membrane and regulate mitochondrial functions (Foster et al., 2012; Laskowski et al., 2019). While several mitochondrial proteins (Sdhb, Uqcrq, CoxIV and Atp5a1) did not change by western blot (Supplementary Figure 2F), we looked for changes in expression of genes encoding additional subunits of the respiratory chain, mitochondrial protein homeostasis, and dynamics (Table 1). However, consistent with the western blot analysis, the mitochondrial genes examined did not change significantly, except for Mfn1, which exhibited a modest, yet significant, downregulation.

Signaling through the mTORC1/Raptor pathway is essential for cerebellum development, and disruption of this pathway in PCs and granule cells leads to a range of developmental abnormalities, from incomplete development and early lethality to long-term deficits in motor coordination and behavior (Tsai et al., 2012; Nakamura et al., 2017; Wu et al., 2017). In parallel, mTORC2/Rictor signaling has also emerged as a critical regulator of cerebellar neuronal function and behavior (Thomanetz et al., 2013; Angliker et al., 2015). We therefore examined the expression of genes encoding components of the mTOR signaling pathway in PKO cerebellum. We found reduced expression of the negative upstream regulators Tsc1, Tsc2 and the mTORC1 subunit Raptor, while genes encoding downstream effectors (Eif4e, Rps6ka3) were unaffected (Table 1). Moreover, the mTORC2 component Rictor was also transcriptionally unchanged.

In summary, our targeted gene expression analysis presented above identified changes in functionally distinct groups (genes associated with specific cerebellar neuronal subtypes, K^+^ channel subunits, and mTOR signaling mediators), providing insight into the molecular consequences of ROMK loss in cerebellar circuits.

### Assessment of oxidative stress–related gene expression changes in PKO cerebellum

It has been reported that ROMK mitigates cell death arising from reactive oxygen species (ROS) overload (Foster et al., 2012). To test whether lack of ROMK in PKO cerebellum elicits compensatory gene expression responses related to ROS production, we used an RT^2^-Profiler PCR array, surveying > 80 ROS-related gene targets, (production, sensing and detoxification, Supplementary Figure 5; Supplementary Table 4). Principal component analysis (PCA, Supplementary Figure 5B) and hierarchical clustering heatmap (Supplementary Figure 5C) revealed that the Control and PKO samples largely overlapped in the expression profiles of the tested genes, given the variability observed within each group (Supplementary Figure 5C; Supplementary Table 4). From this exploratory analysis, we identified a set of 9 genes trending to be downregulated in PKO cerebellum (*p*-value 0.05–0.1; Supplementary Table 4). The list of these genes includes Peroxiredoxin 6 (Prdx6), Neuroglobin (Ngb), Superoxide dismutase 2 (Sod2), Sulfiredoxin 1 (Srxn1), Glutathione s-transferase p (Gstp), NADPH oxidase organizer 1 (Noxo1), Copper chaperone for superoxide dismutase (Ccs) and Prion protein (Prnp, Supplementary Figure 5D). Moreover, the gene Glyceraldehyde-3-phosphate dehydrogenase (Gapdh), often serving as housekeeping reference gene, showed a trend for downregulation (Supplementary Figure 5D). Interestingly, this analysis did not find any of the tested genes to be upregulated. Taken together, these findings suggest that loss of ROMK does not elicit a robust oxidative stress response in the cerebellum, though subtle transcriptional downregulation of select antioxidant and redox-related genes may indicate mild alterations in oxidative homeostasis.

### PKO cerebellum shows elevated expression of the glial marker GFAP

**Figure 4:**
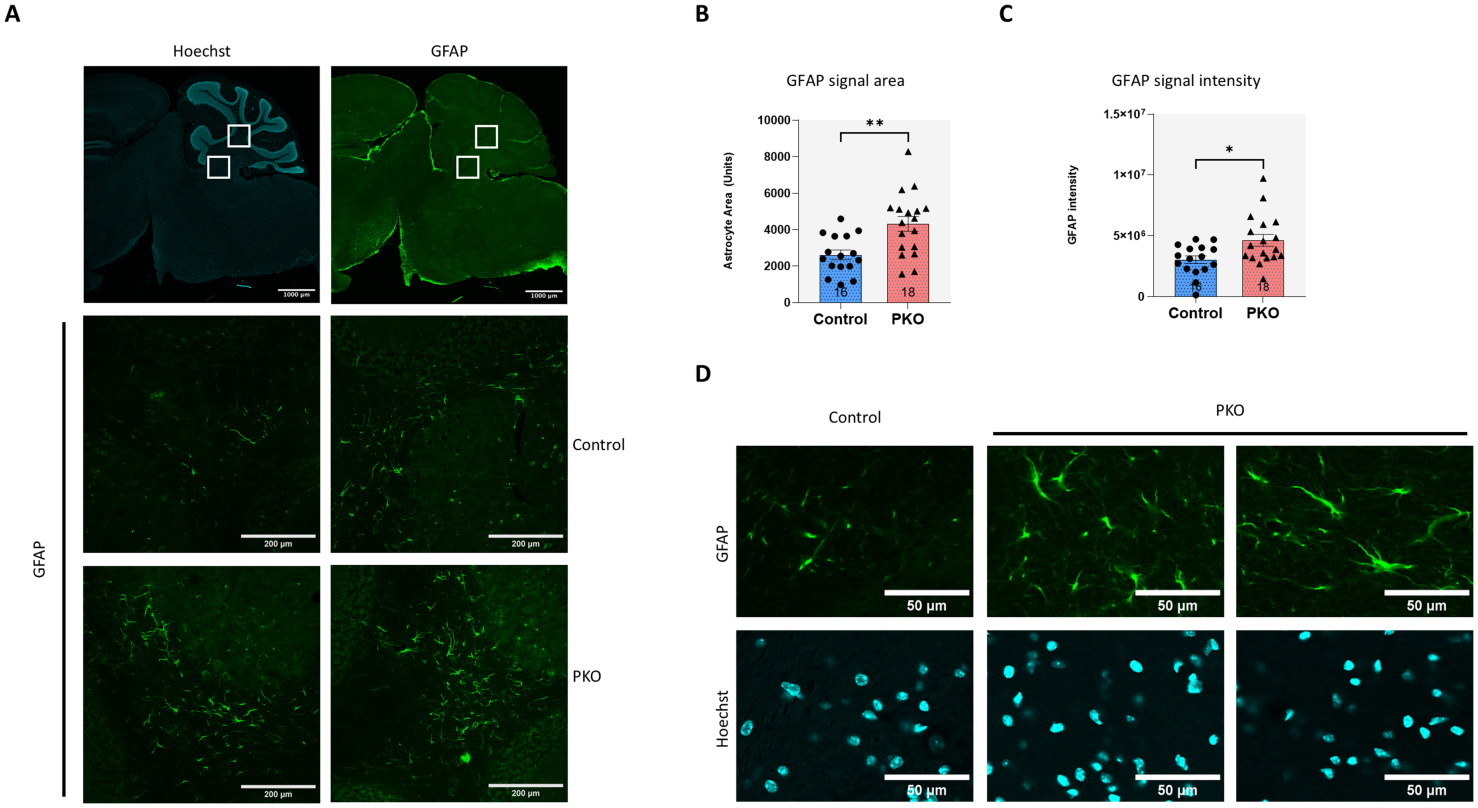
Glial fibrillary acidic protein (GFAP) staining highlights enhanced astrocyte signal in PKO versus control cerebellum. **(A)** Low magnification sagittal sections of control and PKO brain stained for GFAP and highlighting regions of interest. Representative cerebellum regions stained for GFAP are shown below as indicated in the squares above to indicate increased GFAP signal in the white matter of the cerebellum adjacent to the granule cell layer. **(B,C)** Quantitative analysis of GFAP-stained cerebellar sections, assessing total GFAP-positive area and overall GFAP intensity. Confocal images were acquired at multiple regions using a 20 × objective and analyzed in Fiji/ImageJ using area and integrated density measurements. Bar graphs represent aggregated data from multiple fields of view obtained from control and PKO mice (*n* = 3 per genotype). Statistical comparisons were performed with unpaired two-tailed *t*-test. *p < 0.05, **p < 0.001. **(D)** Higher magnification confocal images (40 × objective) of GFAP-stained cerebellar sections from ontrol and PKO mice, highlighting zoomed-in regions of the granule cell layer. The morphology of GFAP-positive cells, exhibiting star-shaped soma and fine branching processes, is consistent with increased cerebellar astrocytes in PKO brains.

Previous studies have implicated glial responses, including astrocyte activation, in mediating or amplifying cerebellar dysfunction in various neurological models (Khakh and Sofroniew, 2015; Cerrato, 2020). GFAP (glial fibrillary acidic protein) upregulation is a hallmark of reactive gliosis (Pekny and Pekna, 2016), and it has been associated with disrupted neuronal signaling and cerebellar dysfunction (Shibuki et al., 1996; Kojic et al., 2018; Gyengesi et al., 2019; Tyszkiewicz et al., 2021). We therefore examined GFAP immunoreactivity in PKO cerebellum to assess whether ROMK deficiency elicits glial activation (Figure 4A). Indeed, we observed significant increase in GFAP signal in the PKO cerebellum, both in terms of overall area taken by GFAP-positive cells and overall GFAP signal intensity (Figures 4B,C). The appearance of GFAP-positive cells was typical of resident cerebellar astrocytes (Figure 4D) and these cells were primarily found in regions adjacent to the granule layer and the cerebellar white matter, rather than within the molecular layer (boxed regions, Figure 4A). The increased area and intensity of GFAP-positive astrocytes, together with their localization are consistent with reactive astrogliosis, potentially reflecting an ongoing pathological response in the PKO cerebellum.

Because Kir4.1 supports astrocytic K^+^ buffering and is often evaluated alongside markers of astrogliosis such as GFAP, we assessed Kir4.1 in cerebellar lysates (Supplementary Figure 4D). Kir4.1 immunoblot showed the expected multiband pattern, however normalized densitometry revealed no significant differences between Control and PKO across the monomer, higher-mass range, or total-lane signal (Supplementary Figures 4E–G). Thus, under our assay conditions, increased GFAP in PKO cerebellum was not accompanied by a detectable bulk change in Kir4.1.

### PKO mice show impaired motor coordination

**Figure 5:**
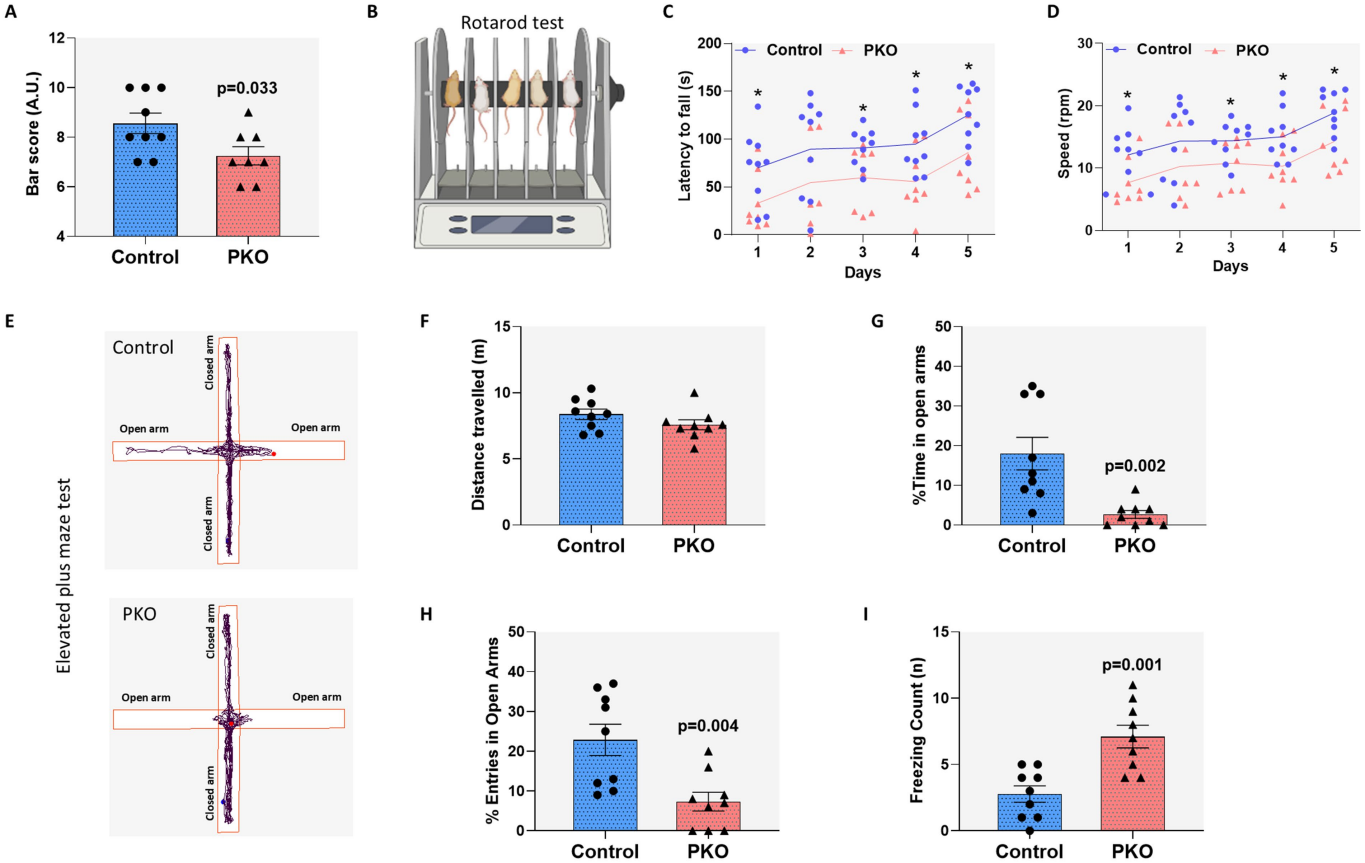
Behavioral assessment shows significant impairment in balance coordination and an anxiety-like behavior in PKO mice. **(A)** The horizontal bar test results represent the Bar score in arbitrary units (A. U). Comparative analysis between control (*n* = 9) and PKO (*n* = 8) mice was conducted using a two-tailed *t*-test. **(B)** A schematic illustration of the rotarod test. Tests were performed following acclimatization of the mice and measurements were taken over 5 consecutive days at 11:00 a.m. The study was performed using male mice, aged 5–8 months old. **(C)** Assessment of ‘Latency to fall’ expressed in seconds (s) after initiation of the rotarod test. The experimental cohort consisted of 9 control and 8 PKO mice. Comparisons between the two groups were performed using repeated measures ANOVA and Tukey *post-hoc* test. *p < 0.05. **(D)** The average rotarod speed in revolutions per minute (rpm) for both groups over 5 consecutive days, analyzed with repeated measures ANOVA and Tukey *post-hoc* test. **(E)** Schematic representations of the elevated plus maze behavioral test in control and PKO mice. Data were recorded using a digital camera placed above the maze. These representative results show that control mice exhibit prolonged presence in the open arms compared to PKO mice. **(F)** Overall distance traveled during the elevated plus maze behavioral test in control (*n* = 9) and PKO (*n* = 9) mice. There is no statistically significant difference between the two groups. **(G)** The percentage of time spent in open arms shows significantly reduced open arm exploration in PKO mice. **(H)** The percentage of open arm entries reveals significantly fewer entries into open arms by PKO mice. **(I)** Freezing counts per mouse during the elevated plus maze test, indicating significantly higher freezing behavior in PKO mice compared to controls. The experimental cohort in the data (panels **F–I**) consisted of 9 control and 9 PKO mice. Comparisons between the two groups were performed using unpaired Student’s *t*-test.

The cerebellum is chiefly involved in motor control such as movement coordination, balance, and posture (Jörntell, 2017), and the axons of PCs are the sole output for the cerebellar cortex, thus being quintessential for motor coordination (Jelitai et al., 2016). K^+^ channels shape cerebellar excitability and signaling both directly, through cell-intrinsic mechanisms, and indirectly, by influencing neighboring cells and contributing to extracellular K^+^ buffering (Millar et al., 2000; Etzion and Grossman, 2001; Kofuji and Newman, 2004; Khavandgar et al., 2005), and mutations or aberrant expression of K^+^ channel genes are often associated with motor coordination deficits (Hoxha et al., 2018). On these grounds, we compared motor coordination performances between Control and PKO mice, with a focus on balance. In the triple horizontal bars test, PKO mice scored lower than WT littermates (Figure 5A, *p* < 0.05). These findings were corroborated by the rotarod test, a gold standard for evaluating motor coordination. Rotarod tests were carried out over a period of five consecutive days. Starting from the first day, PKO mice showed impaired rotarod balance and in the following 4 days, their performance was consistently lower than that of control littermates (Figures 5B–D) both in terms of latency to fall from the rotarod and maintaining balance at higher speeds.

### PKO mice exhibit increased anxiety-like behavior

Beyond its role in motor coordination, the cerebellum plays an important role in cognitive and emotional processes (Levisohn et al., 2000; Sathyanesan et al., 2019), including associative learning and anxiety regulation (Moreno-Rius, 2018; Chin and Augustine, 2023). Furthermore, cerebellar neuronal dysfunction has been linked to anxiety and abnormalities in social behavior (Peter et al., 2016; Yang et al., 2022; Zanin et al., 2023). Accordingly, we examined parameters of anxiety-like behavior in PKO mice using the elevated plus maze (EPM). In this test, the total distance traveled was similar between control and PKO mice, indicating preserved movement through the maze in PKO (Figures 5E,F). However, when parameters classically associated to anxiety-like behavior were examined, PKO mice showed marked alterations. PKO mice spent 85% less time in the open arms compared with control mice (Figure 5G, *p* = 0.002) and made 70% fewer entries in the open arms (Figure 5H, *p* = 0.004). In addition, PKO mice exhibited a strong increase in freezing counts (Figure 5I, *p* = 0.001).

### PKO mice exhibit normal renal ROMK expression and kidney function

ROMK has a well-established role in kidney function, and systemic renal dysfunction and electrolyte imbalance in the event of off-target ablation could secondarily influence performance in behavioral assays. It was therefore important to confirm that the genetic strategy employed here did not result in unexpected effects on the kidney due to a potentially leaky Pcp2cre expression. Examination of tdTomato fluorescence in Pcp2cre-expressing mice did not identify any widespread off-target recombination in the kidney (Supplementary Figure 6A), although a low degree of signal was detected in punctate formations in the kidney cortex (Supplementary Figure 6B). Importantly, there were no significant differences in ROMK expression between Control and PKO kidneys, as assessed by immunofluorescent ROMK staining (Supplementary Figures 6C–E). Likewise, there were no significant changes in overall kidney mass normalized to body mass (Supplementary Figure 6F), a typical defect observed in mice deficient for ROMK in the kidney (Papanicolaou et al., 2020). Kidney damage in mice can often result in changes in the amount of proteins present in the urine. We therefore examined the amount of protein present in the urine of Control and PKO mice. This analysis did not reveal any drastic differences in urinary protein content between the two groups (Supplementary Figures 6G,H). Taken together, this examination suggests normal kidney function in PKO mice, supporting the interpretation that there is no leaky cre-mediated targeting of ROMK in this organ that could be confounding the phenotype of PKO mice.

## Discussion

Understanding the molecular mechanisms underlying ion channel function in neurons has been the subject of extensive research in behavioral neuroscience (Imbrici et al., 2013). In humans, there are 77 genes responsible for encoding subunits of K^+^ channels, making it the most extensive and varied family of ion channels (Coetzee et al., 1999). Owing to this diversity, further investigation is required to completely unravel the functions of K^+^ channels in neuronal activity. In particular, the role of ROMK (Renal Outer Medullary K^+^) channels has been primarily investigated in renal function and cardiovascular physiology. However, the recent development of conditional ROMK mouse models (Schiano et al., 2019; Papanicolaou et al., 2020), allows studying the involvement of ROMK channels in other tissues, including the central nervous system. In this work, we used a cre-mediated gene knockout approach to examine the potential role of ROMK in cerebellar neurons, in motion coordination and anxiety-like behavior. We found that: (1) ROMK is expressed in the cerebellum, in cells forming localized areas within the granule layer but not in the Purkinje or molecular layers. (2) Loss of ROMK does not grossly impact PC density and PC soma size, although it promotes increased PC dendrite diameter, (3) Broad interrogation of molecular signaling pathways and gene expression patterns in the PKO cerebellum revealed reduced expression of neuronal subtype-specific genes and K^+^ channel family genes, (4) there was evidence of astrocyte activation in PKO cerebellum, and (5) PKO mice exhibited impaired motor coordination and elevated anxiety-like behavior in the EPM. Collectively, the present findings identify a functional role of ROMK in the cerebellum and implicate ROMK in behavioral dysfunction.

### Spatial and cellular characteristics of ROMK ablation in the PKO model

To date, most studies have focused on ROMK expression and function in the kidney, where it plays a crucial role in maintaining K^+^ balance (Welling, 2016). However, additional evidence suggests that ROMK channels are also expressed in the brain (Shuck et al., 1994; Kondo et al., 1996; Foster et al., 2012). To genetically dissect the roles of ROMK in the brain, we used the neuronal Pcp2cre driver (Barski et al., 2000). Mapping of cre activity with the tdTomato reporter revealed that cre was present in Purkinje cells, but also in cells of the granule layer. ROMK itself was not detected in Purkinje cells; instead, ROMK immunoreactivity appeared as a punctate pattern within the granule layer and was significantly reduced in PKO. ROMK expression was preserved in other parts of the PKO brain (cortex, brainstem) and remained intact in the kidney. Taken together, these data indicate that our PKO model produces loss of ROMK that is confined to the cerebellum, likely involving granule-layer cells that have low-level Pcp2cre activity.

Given that Pcp2cre activity is not strictly confined to a single cerebellar cell class, cellular attribution in this model should be interpreted cautiously. The aggregate data are compatible with involvement of a ROMK-expressing granule-layer population, but they do not exclude other cerebellar cell types. Future studies using more selective Cre drivers for the granule layer together with spatial, or single-cell focused approaches could help clarify ROMK expression and function in distinct cerebellar populations.

### A proposed working model: disrupted K^+^ handling in cerebellar circuits as the basis of molecular, cellular, and behavioral phenotypes

**Figure 6:**
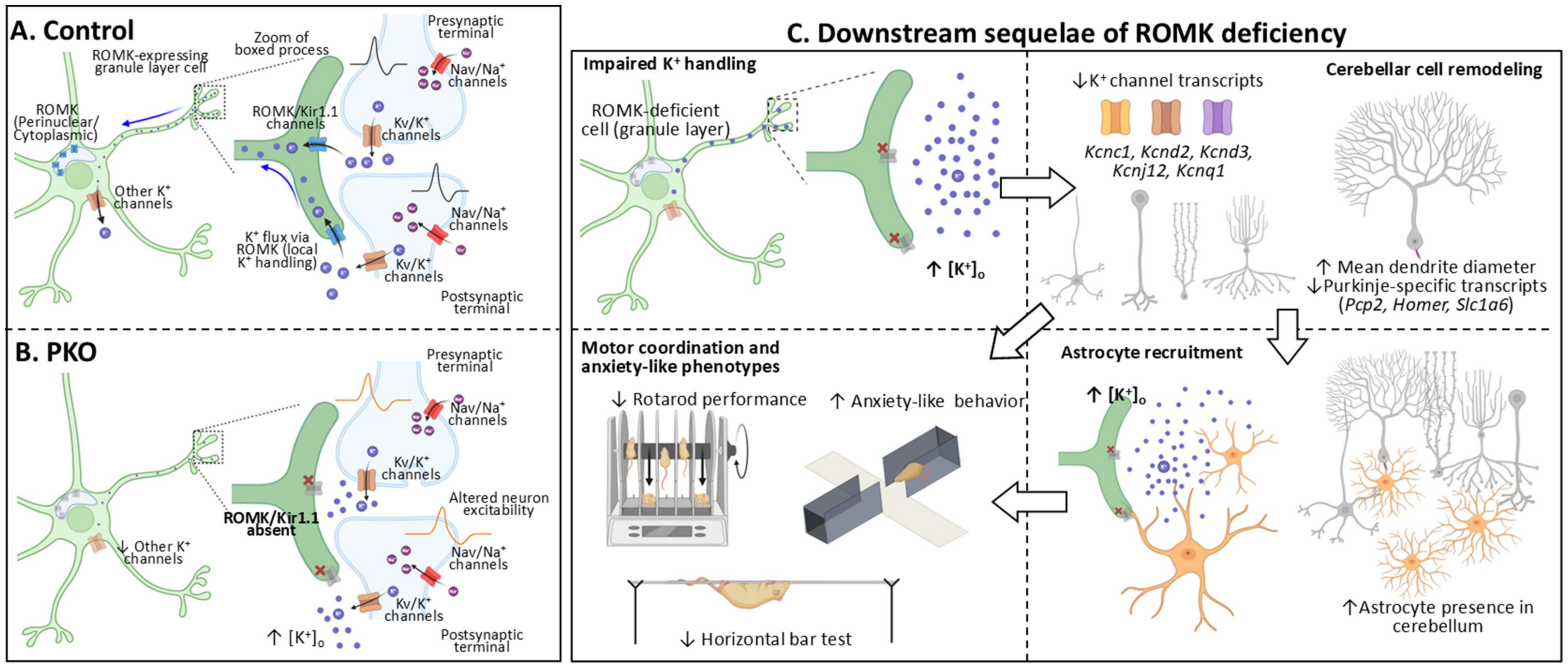
Conceptual working model linking ROMK loss to cerebellar remodeling and behavioral changes. **(A)** Control: ROMK (Kir1.1) is shown expressed in a cerebellar granule layer cell. In this model, ROMK is shown forming channels on a cellular process adjacent to a synapse; the blue arrow indicates a potential pathway for K^+^ flux contributing to K^+^ homeostasis at the synapse. Under these conditions, K^+^ accumulation during high neuronal activity is buffered effectively, and neuronal excitability is preserved. **(B)** PKO: Genetic ablation of ROMK in Pcp2cre-expressing granule layer cells is proposed to impair this K^+^ buffering pathway, resulting in elevated extracellular K^+^ within the local microdomain and altered excitability of neighboring neurons. **(C)** Downstream sequelae: Disruption of K^+^ homeostasis is associated with cerebellar remodeling, including downregulation of multiple K^+^ channel transcripts (*Kcnc1, Kcnd2, Kcnd3, Kcnj12, Kcnq1*, found in different cerebellar cell types), reduced expression of Purkinje-enriched markers (*Pcp2, Homer, Slc1a6*), Purkinje dendritic remodeling, and increased presence of astrocytes in the cerebellum. Together, these cellular changes are proposed to contribute to the motor coordination deficits and anxiety-like behavior of PKO mice. Solid arrows indicate proposed relationships. Source for graphics: BioRender.

As a working model, we propose that ROMK contributes to local K^+^ buffering within discrete synaptic environments of the cerebellum. Its loss may impair the regulation of extracellular K^+^ homeostasis and disrupt the fine-tuned firing properties of cerebellar neurons. Such ionic imbalances may underlie the array of molecular, cellular, and behavioral changes observed in the PKO model (Figure 6). Although ROMK immunoreactivity is predominantly intracellular and perinuclear at the resolution of our imaging, the known properties of inwardly rectifying K^+^ channels suggest that ROMK could provide a low-resistance pathway for K^+^ flux between these cells and the extracellular space (Figure 6A). In this framework, ROMK loss is proposed to impair local K^+^ handling during synaptic activity, resulting in elevated extracellular K^+^ within local microdomains and altered neuronal excitability (Figure 6B). We also speculate that disruption of this mechanism of regional K^+^ homeostasis may elicit compensatory responses, including reduced expression of multiple K^+^ channel genes across cerebellar cell types, and astrocyte recruitment to enhance regional K^+^ handling (Figure 6C). However, chronic alterations may also be associated with Purkinje cell remodeling, including changes in Purkinje dendrites and reduced PC-specific markers, and additional recruitment of astrocytes in the area. We expect that these chronic disruptions culminate in the behavioral defects observed in PKO mice, including impaired motor coordination and anxiety-like behavior (Figure 6C).

### ROMK loss and associated changes in cerebellar K^+^ channel gene expression

As stated earlier, K^+^ channels form a large family of ion channels that establish neuronal resting membrane potential, shape the action potential, and modulate neurotransmitter release (Zagha et al., 2008; Humphries and Dart, 2015; Kaczmarek and Zhang, 2017). Functional analyses indicate that voltage-gated, inwardly rectifying, and leak K^+^ channels serve important roles in cerebellar granule layer neurons (Mathie et al., 2003). Interestingly, in the PKO cerebellum, we found downregulation of voltage-gated K^+^ channels, of different types. These included Kv3 channels (*Kcnc1*), Kv4 channels (*Kcnd2* and *Kcnd3*) and Kv7 channels (*Kcnq1*). We propose that this coordinated reduction in Kv channel expression reflects an adaptive response to recalibrate neuronal excitability under disrupted local K^+^ handling in PKO (Figure 6C). Kcnd2 and Kcnd3 in particular play important roles in shaping action potential in granule cells (Shibata et al., 2000) and mutations in these genes cause spinocerebellar ataxias (Lee et al., 2012; Smets et al., 2015). Kv3 channel expression in cerebellar granule neurons and Purkinje cells is also critical in influencing firing patterns (Joho and Hurlock, 2009).

Within the Kir family (inwardly rectifying K^+^ channels), the Kir2.x channels participate in both setting the resting potential and in late repolarization in neurons (Neusch et al., 2003; Prüss et al., 2005), while G-protein-regulated Kir3.x channels couple receptor activation to PC excitability (Johansson and Hesslow, 2020; Lippiello et al., 2020). ATP-dependent Kir6.x channels, which share some common properties with ROMK, may also regulate neuronal excitability (Clark et al., 2010). Our survey, however revealed that only Kir2.2 (*Kcnj12*) was significantly downregulated, while other Kir family members (*Kcnj2*, *Kcnj3*, *Kcnj5*, *Kcnj6* or *Kcnj9*) were not altered. Finally, we observed a reduction in the BK channel *α*-subunit (encoded by *Kcnma1*) at the protein level in PKO cerebellum, a change that is relevant in our model given that loss of BK channel function can cause cerebellar ataxia and Purkinje cell dysfunction (Sausbier et al., 2004).

Taken together, these findings point to coordinated remodeling of potassium channel expression across multiple families in PKO cerebellum. We propose that these changes may begin as adaptive responses to disrupted regional K^+^ homeostasis within the granule layer, yet over time they could affect excitability and synaptic integration in cerebellar circuits more broadly. In line with this idea, disrupted K^+^ handling and excitability in the granule layer could propagate to Purkinje cells via granule neuron projections into the molecular layer (parallel fibers), providing an indirect route to changes in Purkinje-specific gene expression and dendritic structure over time (Figure 6C).

In contrast to the K^+^ channel transcript analysis, our targeted ROS transcript screen did not reveal a coordinated expression signature in PKO cerebellum. The lack of consistent changes in ROS-handling genes suggests that global oxidative-stress programs are not prominently engaged and argues against a primarily ROS-driven mechanism in this context. These results are therefore consistent with the interpretation that disrupted K^+^ handling is a central feature of the PKO phenotype and the observed cellular and behavioral findings. This interpretation also aligns with the broader view that ROMK-related pathophysiology is most often linked to ion mishandling rather than primary ROS dysregulation.

### Increased presence of GFAP-positive cells in PKO cerebellum and their potential roles in K^+^ buffering and cerebellar homeostasis

In the PKO cerebellum, we observed a notable accumulation of GFAP-positive cells, primarily localized to regions adjacent to the granule layer and white matter. Although not excitable, astrocytes are electrically dynamic, and play crucial roles in maintaining extracellular ion homeostasis, including buffering excess extracellular K^+^ generated during heightened neuronal activity (Kofuji and Newman, 2004; McNeill et al., 2021). The bulk of K^+^ buffering in astrocytes is mediated by Kir4.1 (*Kcnj10*), and in the cerebellum this channel is found expressed in granule layer astrocytes and in Bergmann glia processes (Brasko et al., 2017). However, our assessment did not reveal significant changes of Kir4.1 in the PKO cerebellum. It should be noted that apart from Kir4.1, other Kir channels have been proposed to play ancillary roles to that of Kir4.1 in K^+^ buffering by astrocytes (Brasko et al., 2017; Brasko and Butt, 2018; Hu et al., 2019).

Beyond ion buffering, astrocytes modulate synaptic transmission (Liu et al., 2023), and participate in neuroinflammatory and neurodegenerative processes (Claycomb et al., 2013; Patani et al., 2023; Mockenhaupt et al., 2025), serving either protective roles, or contributing to maladaptive feedforward mechanisms that exacerbate pathology. Accordingly, the increased presence of GFAP-expressing astrocytes in the PKO cerebellum may reflect both an adaptive response aimed at stabilizing local ionic homeostasis under disrupted K^+^ handling and a secondary reaction to ongoing circuit stress/remodeling as the phenotype evolves (Figure 6C). Importantly, aberrant activation of cerebellar astrocytes has been associated with behavioral impairments and ataxias (Cerrato, 2020; Rosa et al., 2022; Edamakanti et al., 2023), suggesting a potential mechanism contributing to the abnormal behavioral phenotypes observed in PKO mice in this study.

Finally, it is interesting to note that ROMK has been reported in glial-like cells outside the CNS (e.g., type I cells in tongue taste buds) (Dvoryanchikov et al., 2009), whereas studies of optic nerve glial preparations did not support a functional contribution for ROMK in that context (Papanikolaou, 2014). Our current data do not resolve cell type-specific ROMK expression in astrocytes and addressing this question will require dedicated follow-up studies beyond the scope of the present work.

### Involvement of ROMK in behavioral defects

The potassium channel blocker tertiapin-RQ, known to inhibit ROMK along with other Kir channels, was observed to trigger anxiety-like behavior and motor impairments in mice. A comparable behavioral response was seen with tertiapin-LQ, a derivative with enhanced selectivity for ROMK, strengthening the hypothesis that ROMK activity contributes to affective regulation (Okada et al., 2020). Another study using cerebellar infusion of tertiapin-LQ reported impaired associative learning responses in ferrets; although, the effect was attributed primarily to Kir3.1 inhibition in PCs (Johansson and Hesslow, 2020). Taken together, these pharmacological studies suggest a role for ROMK in cerebellar-dependent behaviors, although their interpretation is limited by overlapping channel specificity. In this context, our targeted genetic approach offers a valuable and complementary strategy that supports the specific contribution of ROMK to cerebellar function and behavior.

In our model, ROMK deficiency not only impairs classic cerebellar-dependent motor coordination but is also associated with altered performance on anxiety-related measures in the EPM. We note that interpretation of anxiety-related measures from the elevated plus maze (EPM) requires caution in models with motor impairment. Because PKO mice exhibit deficits on rotarod and horizontal bar tasks, reduced open-arm exploration could reflect reduced confidence navigating an elevated/exposed environment, rather than a primary change in affective state. In our dataset, total distance traveled in the EPM was unchanged, arguing against a generalized reduction in movement within this task; however, we cannot exclude the possibility that impaired coordination contributes to open-arm avoidance. Accordingly, we interpret the EPM findings as anxiety-like behavior that may be partially influenced by motor deficits, and we acknowledge that definitive evidence for a primary affective dysfunction will require broader behavioral validation.

Further research will be needed to clarify the cellular mechanisms by which ROMK deficiency alters cerebellar function, and to determine whether ROMK plays related roles in other cell types within the cerebellum, or elsewhere in the brain. Such work should expand our understanding of ROMK’s contribution to ion homeostasis and neural function, offering new insights into how channel dysregulation can influence behavior, with potential relevance for neuropsychiatric and movement disorders.

## Data Availability

The datasets generated and analyzed during the current study are included in the published article and its supplementary material. Additional data supporting the findings of this study are available from the corresponding author upon reasonable request.

## Supplementary material

SUPPLEMENTAL FIGURE 1 Survey of ROMK immunolabeling in hippocampus, cortex, and brainstem. **(A)** Allen Mouse Brain Atlas reference map (sagittal orientation) with regional labels (for abbreviations see below). Low magnification sagittal views of Control (middle) and PKO (right) brains with boxed areas showing (i) hippocampus and (ii) cortex that correspond to imaging regions below. **(B)** Hippocampus in Control and PKO brains. Representative low-magnification images (upper row; scale bar, 1,000 μm) and higher-magnification views (lower row; scale bar, 50 μm). Under these conditions, ROMK signal in hippocampal fields was at or near background in both genotypes, and no discrete ROMK labeling was observed. **(C)** Cortex at sagittal levels that include the Superior colliculus (SC). High-magnification images (scale bar, 50 μm) show ROMK-positive cells (white arrows) present at similar frequencies in both Control and PKO brains. **(D)** Representative immunohistochemistry in the brainstem of either Control or PKO brains identifies ROMK-positive cells (arrows) with similar frequencies in the two groups. To obtain these images, sagittal sections (50 μm, free-floating) from Control and PKO brains were processed as described in methods using ROMK antibody (Proteintech, Cat. No. 20953-1-AP) and followed by tyramide signal amplification (Thermo Fisher Alexa Fluor 488 Tyramide SuperBoost Kit, Cat. B40922). Sections were subsequently stained with Hoechst nuclear staining, mounted and imaged on an Olympus FV3000RS confocal microscope. Laser power, detector gain, and exposure were held constant across genotypes and brain regions. Select abbreviations for panel A: HPF, hippocampal formation; CA1–CA3/CA4, Cornu Ammonis fields 1–3/4; DG, dentate gyrus; SUB, subiculum; POST, postsubiculum; RSPd, dorsal retrosplenial area; alv, alveus; so/sp/sr, strata oriens/pyramidale/radiatum; DG-mo, Dentate gyrus, molecular layer; DG-sg, Dentate gyrus, granule cell layer; DG-po, Dentate gyrus, polymorph layer; LP, lateral posterior thalamic nucleus; PO, posterior thalamic nucleus; SCm, superior colliculus, motor-related.

SUPPLEMENTAL FIGURE 2 Normal molecular signaling markers in PKO cerebellum. **(A)** Western blot analysis of cerebellum from Control and PKO mice to assess the expression of the Tyrosine kinase receptor B (TrkB) and Brain-Derived Neurotrophic Factor (BDNF) running as a multimer at 30 kDa. Total protein staining was used to normalize for protein loading. **(B,C)** Band densitometry for TrkB and BDNF proteins, respectively, in Control and PKO cerebella. ns: not significant difference. **(D)** Quantitative real-time PCR analysis of Bdnf mRNA expression in Control and PKO cerebellum. The expression was normalized to the expression of Beta-2 Microglobulin (B2M) housekeeping gene. The numbers in the bar graphs indicate the number of different cerebella analyzed per group. **(E)** Western blot analysis from Control and PKO cerebella, to assess the phosphorylation status of signaling mediators Erk1/2 (Extracellular Signal-Regulated Kinase 1/2) and Creb (cAMP Response Element-Binding protein). **(F)** Assessment of the abundance of mitochondrial proteins serving as subunits of the Electron Transport Chain (ETC) and ATP synthase. Markers examined were the Sdhb (Succinate Dehydrogenase Complex Iron Sulfur Subunit B), Uqcrq (Ubiquinol-Cytochrome c Reductase Complex III Subunit VII), CoxIV (Cytochrome c Oxidase Subunit IV), and Atp5a1 (ATP Synthase Subunit Alpha). **(G)** Assessment of autophagy markers in the same cerebellar samples. The analysis includes the expression of Ulk1 (Unc-51 Like Autophagy Activating Kinase 1), Sqstm (Sequestosome 1), and LC3B (Microtubule-Associated Protein 1A/1B-Light Chain 3B).

SUPPLEMENTAL FIGURE 3 Increased abundance of S6 ribosomal protein in PKO cerebellum. **(A)** Low magnification (x2) confocal images of cerebellum coronal sections stained with the PC marker Calbindin (green), ribosomal protein S6 (red) and counterstained with Hoechst (blue). The scale bar in these images is 2000 μm. **(B)** Region of the above scanned at a higher magnification (x40) showing that the majority of rpS6 signal localizes within the PCs and a smaller fraction in the granule layer. The images were reconstructed using maximum z stack projection to obtain detailed images of PCs and other cells in the cerebellum. The scale bar is 100 μm. **(C,D)** Western blot analysis to evaluate the abundance of the ribosomal protein S6 in Control and PKO cerebellum. The study utilized 4 samples per group, and statistical comparisons were performed using unpaired student’s *t*-test. ***p* < 0.01.

SUPPLEMENTAL FIGURE 4 Reduced protein levels of the big K^+^ channel subunit Kcnma1 in PKO cerebellum. **(A–C)** Western blot analysis in whole cerebellum lysates from Control and PKO samples (*n* = 4 biological replicates per group, 20 μg total protein loaded per lane) to assess the expression of ion channels Kcnma1 (Calcium-Activated Potassium Channel Subunit Alpha-1, also known as MaxiK or BK channel) and Cftr (Cystic Fibrosis Transmembrane Conductance Regulator). Band intensities corresponding to Kcnma1 were normalized to total protein loading and comparisons between Control and PKO samples were carried out using the unpaired Student’s *t*-test. **p* < 0.05. **(D)** Western blot analysis of Kir4.1 in Control and PKO cerebellum. Brackets show expected Kir4.1 monomer ~ 35–40 kDa and higher-mass species ~ 140-180 kDa. **(E–G)** Normalized densitometry at the 35–40 kDa range **(E)**, the 140-180 kDa range **(F)**, and the total lane signal (30–250 kDa; including mid-MW bands at ~45–100 kDa) **(G)**. Quantification was performed following normalization of each species to their respective total protein loading. Comparisons between Control and PKO samples were carried out using the unpaired Student’s t-test. The differences between Control and PKO groups are not significant (*p* = 0.11, 0.09, and 0.11 for panels **E**, **F**, and **G**, respectively).

SUPPLEMENTAL FIGURE 5 Comparative expression analysis of oxidative stress genes in Control and PKO cerebellum. **(A)** Schematic of the experiment done to analyze the expression of oxidative stress genes in Control and PKO cerebellum. Total RNA was extracted using the Trizol reagent. 1280 ng of purified RNA was used to synthesize cDNA using the RT2 First Strand Kit. The resulting cDNA from each sample was combined with the RT2 SYBR Green qPCR mastermix and applied to 384-well plates pre-loaded with optimized primer pairs for Mouse Oxidative Stress genes, including negative controls and housekeeping genes (PAMM-065ZE). Real-time qPCR was performed on a CFX3984 system, and Ct values were obtained and normalized to that of B2M. Expression values for each gene per sample were log2 transformed and further analyzed statistically in R using the Linear Models for Microarray and RNA-seq data (limma) package and the Benjamini-Hochberg to control the false discovery rate during multiple hypothesis testing. **(B)** Principal Component Analysis (PCA) Plot was performed in R, using packages plotMDS (multidimensional scaling plot) and ggplot. Each data point corresponds to an individual sample, and 3 biological replicates per group (Rep_1–Rep_3) were included in the analysis. **(C)** Construction of the heatmap was performed in R using the package heatmaply. Gene expression values for each gene representing each row were z-scored by subtracting the row mean and dividing by the row standard deviation. The heatmap displays the relative expression levels of Mouse Oxidative Stress genes across Control and PKO cerebellum samples (89 genes total). The clustering was adjusted so that three major clusters of genes are defined as shown by the blue, green and magenta dendrograms. The intensity scale uses color-coded gradients, with warmer colors representing higher expression and cooler colors indicating lower expression levels. **(D)** Heatmap of selected oxidative stress genes from the PCR array that showed a trend for differential expression between Control and PKO cerebellum. The shortlist of oxidative stress genes includes Peroxiredoxin 6 (Prdx6), Neuroglobin (Ngb), Superoxide Dismutase 2 (Manganese) (Sod2), Glyceraldehyde-3-Phosphate Dehydrogenase (Gapdh), Sulfiredoxin 1 (Srxn1), Glutathione S-Transferase Pi 1 (Gstp1), NADPH Oxidase Organizer 1 (Noxo1), Copper Chaperone For Superoxide Dismutase (Ccs), and Prion Protein (Prnp).

SUPPLEMENTAL FIGURE 6 Minimal tdTomato fluorescence in Pcp2cre kidney and normal ROMK expression and renal phenotype in PKO mice. **(A)** Macroscopic images of kidneys from tdTomato^KI^;- and tdTomato^KI^; Pcp2cre^Tg^ mice shown in bright field and fluorescence using a fluorescent stereoscope. **(B)** Left: Fluorescent image of tdTomato^KI^; Pcp2cre^Tg^ kidney sectioned along the long axis to reveal the cortex and medulla, imaged with a confocal microscope (Olympus FV3000, Objective 4x). The scale bar is 1500 μm. Right: A zoomed region encompassing the cortex and medulla, shown at higher magnification. The scale bar in the right micrograph is 500 μm. Low but detectable tdTomato fluorescence above background can be observed in punctate formations in the cortex and elongated formations in the medulla, potentially indicating local Pcp2cre-expressing cell populations in the kidney. **(C)** Immunofluorescence (IF) staining of renal sections from Control and PKO mice showing polarized luminal expression pattern with tubular appearance, consistent with ROMK localization in renal epithelial cells. The imaging was performed using confocal microscopy with the 40× objective. **(D,E)** Confocal images were analyzed using Fiji/ImageJ to determine total ROMK expression in Control and PKO kidneys using area and integrated intensity measurements. Bar graphs represent aggregate data from multiple regions obtained from Control and PKO kidneys (*n* = 3 mice per genotype). No statistically significant differences were observed between groups (unpaired Student’s *t*-test). **(F)** Assessment of whole kidney mass normalized to body weight, as an index of kidney injury in Control and PKO mice (n=8 mice per group). No statistically significant difference in kidney mass was observed between the two groups (unpaired Student’s *t*-test). **(G,H)** Assessment of kidney function via total urinary protein analysis in Control and PKO mice. Urine was collected non-invasively, and samples were analyzed by reducing SDS-PAGE and western blot. Lane intensity was quantified by densitometry and compared between groups (panel H). No statistically significant difference in urinary protein content was detected between Control and PKO mice (unpaired Student’s *t*-test; *n* = 8 Control, 7 PKO).
