# Supplementary figures and images for: Characterizing molecular and behavioral changes arising from ROMK potassium channel deficiency in the cerebellum
Source: Front Behav Neurosci. 2026 Jan 26;19:1681149. doi: 10.3389/fnbeh.2025.1681149 (PMC12883771; doi:10.3389/fnbeh.2025.1681149)

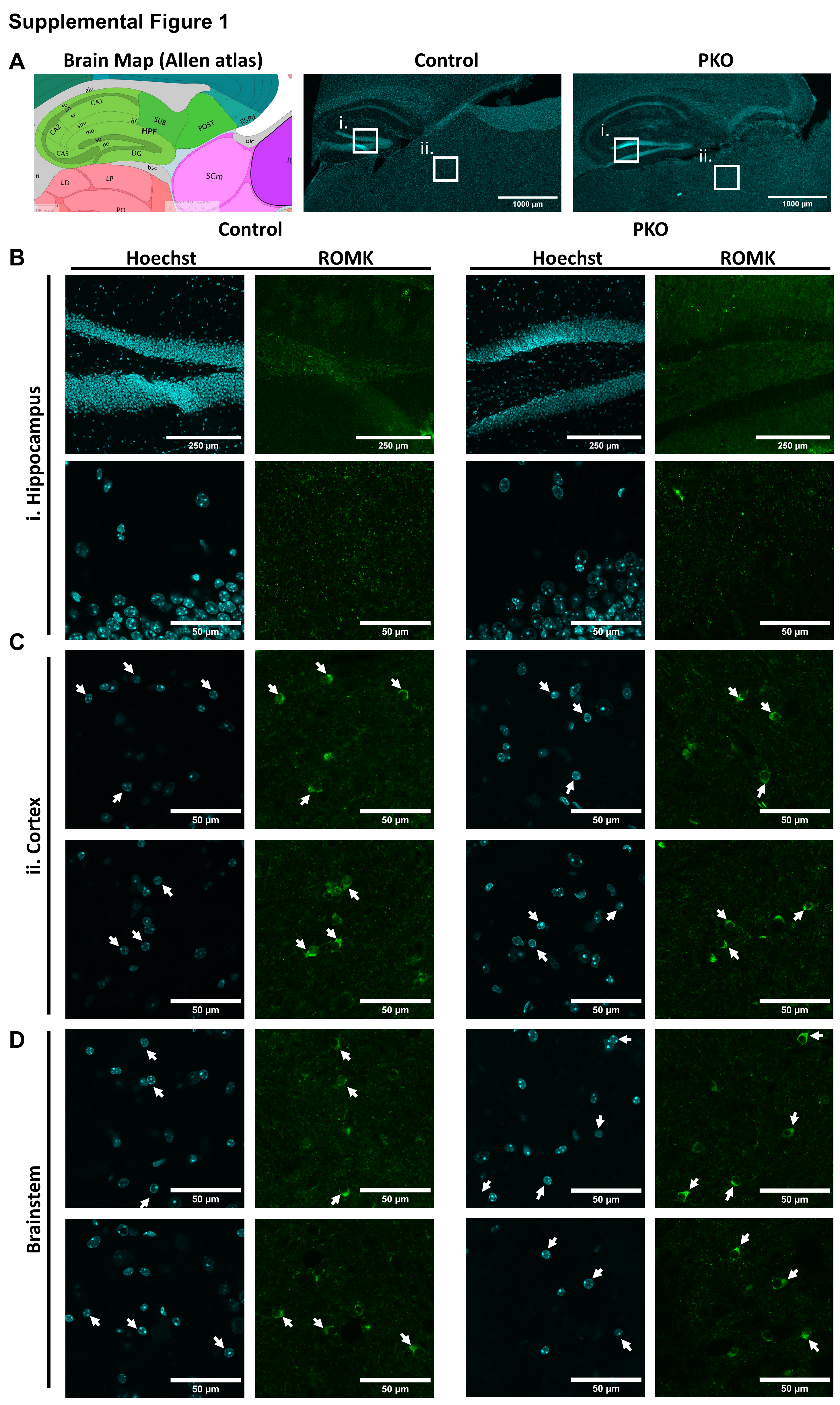

Supplement: SUPPLEMENTAL FIGURE 1 — Survey of ROMK immunolabeling in hippocampus, cortex, and brainstem. (A) Allen Mouse Brain Atlas reference map (sagittal orientation) with regional labels (for abbreviations see below). Low magnification sagittal views of Control (middle) and PKO (right) brains with boxed areas showing (i) hippocampus and (ii) cortex that correspond to imaging regions below. (B) Hippocampus in Control and PKO brains. Representative low-magnification images (upper row; scale bar, 1,000 μm) and higher-magnification views (lower row; scale bar, 50 μm). Under these conditions, ROMK signal in hippocampal fields was at or near background in both genotypes, and no discrete ROMK labeling was observed. (C) Cortex at sagittal levels that include the Superior colliculus (SC). High-magnification images (scale bar, 50 μm) show ROMK-positive cells (white arrows) present at similar frequencies in both Control and PKO brains. (D) Representative immunohistochemistry in the brainstem of either Control or PKO brains identifies ROMK-positive cells (arrows) with similar frequencies in the two groups. To obtain these images, sagittal sections (50 μm, free-floating) from Control and PKO brains were processed as described in methods using ROMK antibody (Proteintech, Cat. No. 20953-1-AP) and followed by tyramide signal amplification (Thermo Fisher Alexa Fluor 488 Tyramide SuperBoost Kit, Cat. B40922). Sections were subsequently stained with Hoechst nuclear staining, mounted and imaged on an Olympus FV3000RS confocal microscope. Laser power, detector gain, and exposure were held constant across genotypes and brain regions. Select abbreviations for panel A: HPF, hippocampal formation; CA1–CA3/CA4, Cornu Ammonis fields 1–3/4; DG, dentate gyrus; SUB, subiculum; POST, postsubiculum; RSPd, dorsal retrosplenial area; alv, alveus; so/sp/sr, strata oriens/pyramidale/radiatum; DG-mo, Dentate gyrus, molecular layer; DG-sg, Dentate gyrus, granule cell layer; DG-po, Dentate gyrus, polymorph layer; LP, lateral posterior [file Image_1.TIF]

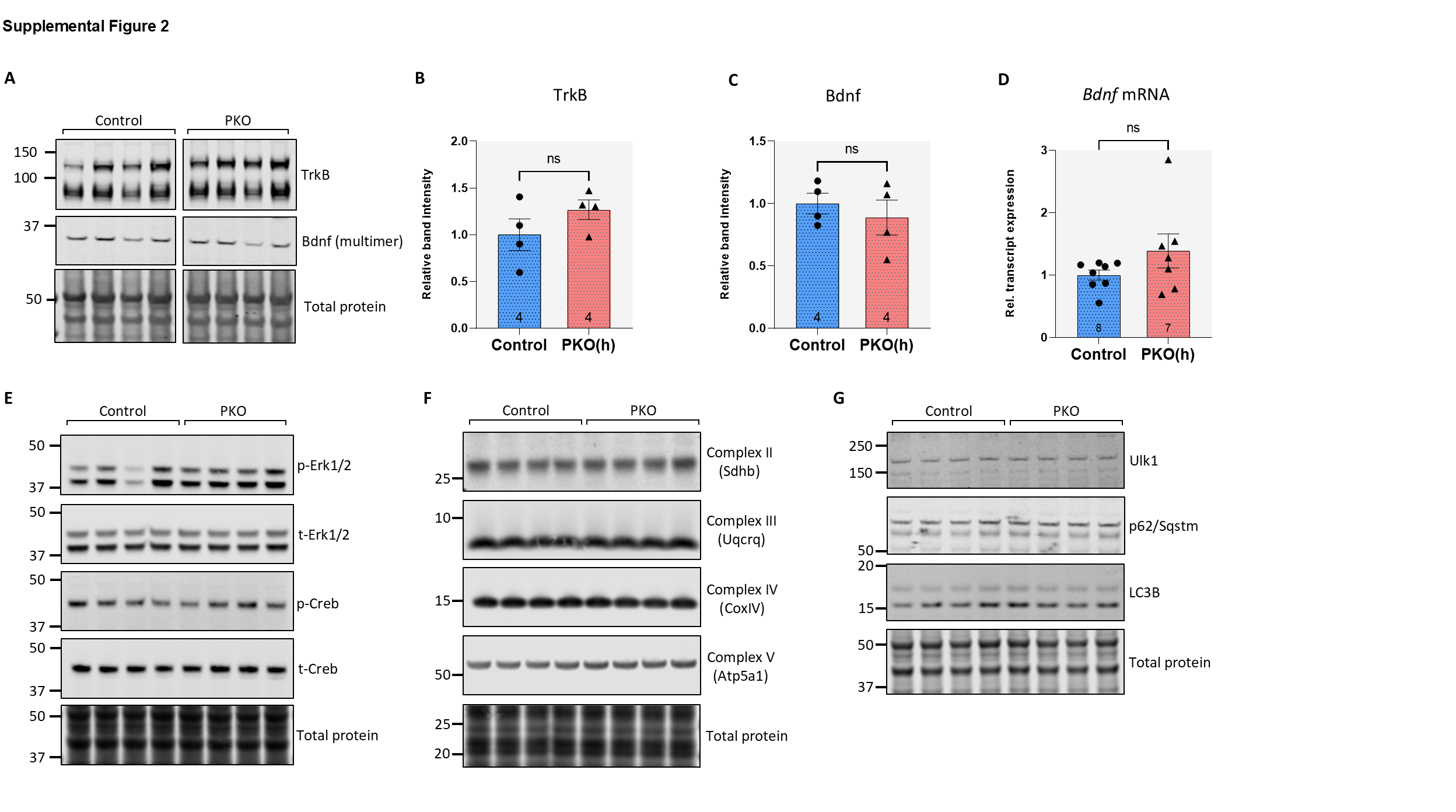

Supplement: SUPPLEMENTAL FIGURE 2 — Normal molecular signaling markers in PKO cerebellum. (A) Western blot analysis of cerebellum from Control and PKO mice to assess the expression of the Tyrosine kinase receptor B (TrkB) and Brain-Derived Neurotrophic Factor (BDNF) running as a multimer at 30 kDa. Total protein staining was used to normalize for protein loading. (B,C) Band densitometry for TrkB and BDNF proteins, respectively, in Control and PKO cerebella. ns: not significant difference. (D) Quantitative real-time PCR analysis of Bdnf mRNA expression in Control and PKO cerebellum. The expression was normalized to the expression of Beta-2 Microglobulin (B2M) housekeeping gene. The numbers in the bar graphs indicate the number of different cerebella analyzed per group. (E) Western blot analysis from Control and PKO cerebella, to assess the phosphorylation status of signaling mediators Erk1/2 (Extracellular Signal-Regulated Kinase 1/2) and Creb (cAMP Response Element-Binding protein). (F) Assessment of the abundance of mitochondrial proteins serving as subunits of the Electron Transport Chain (ETC) and ATP synthase. Markers examined were the Sdhb (Succinate Dehydrogenase Complex Iron Sulfur Subunit B), Uqcrq (Ubiquinol-Cytochrome c Reductase Complex III Subunit VII), CoxIV (Cytochrome c Oxidase Subunit IV), and Atp5a1 (ATP Synthase Subunit Alpha). (G) Assessment of autophagy markers in the same cerebellar samples. The analysis includes the expression of Ulk1 (Unc-51 Like Autophagy Activating Kinase 1), Sqstm (Sequestosome 1), and LC3B (Microtubule-Associated Protein 1A/1B-Light Chain 3B). [file Image_2.TIF]

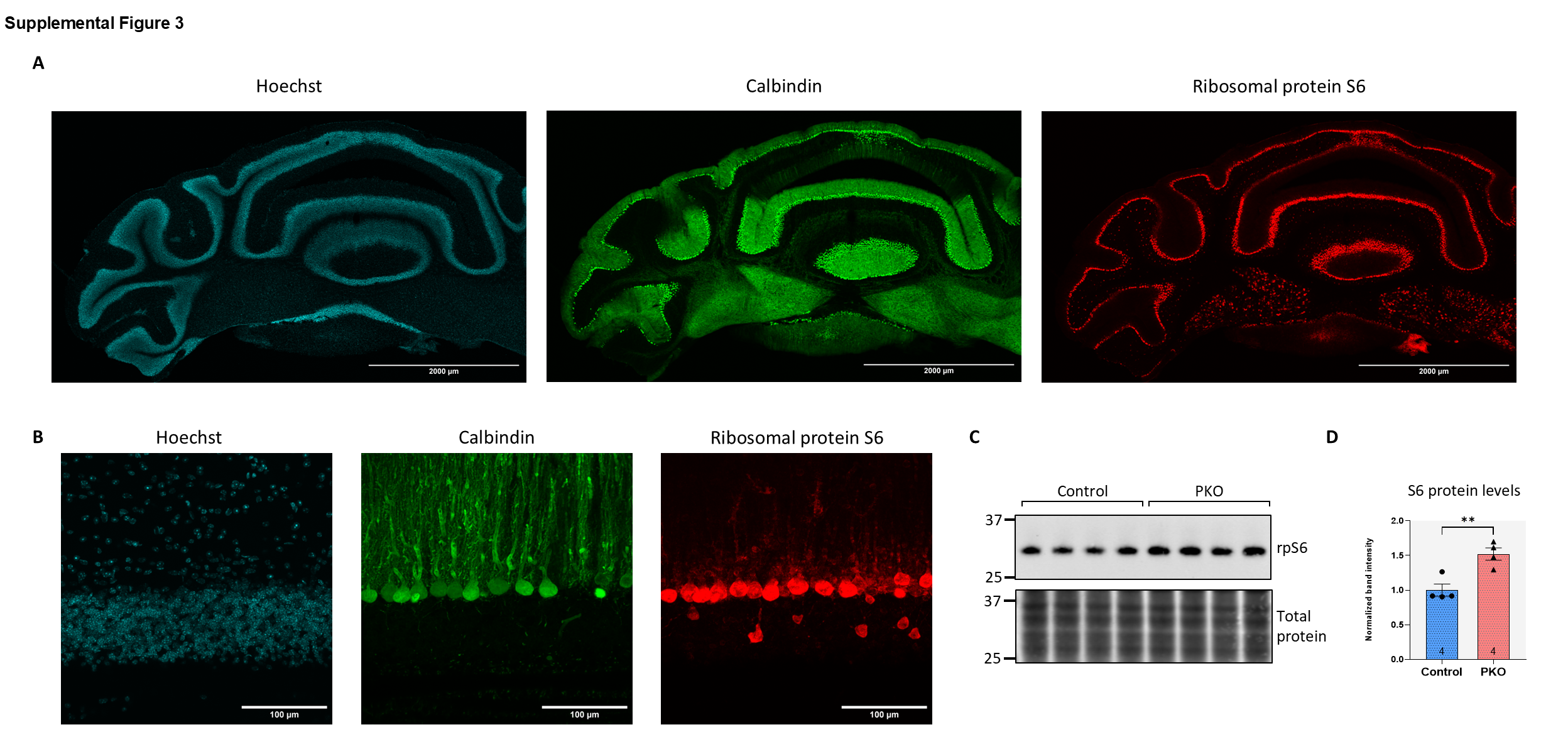

Supplement: SUPPLEMENTAL FIGURE 3 — Increased abundance of S6 ribosomal protein in PKO cerebellum. (A) Low magnification (x2) confocal images of cerebellum coronal sections stained with the PC marker Calbindin (green), ribosomal protein S6 (red) and counterstained with Hoechst (blue). The scale bar in these images is 2000 μm. (B) Region of the above scanned at a higher magnification (x40) showing that the majority of rpS6 signal localizes within the PCs and a smaller fraction in the granule layer. The images were reconstructed using maximum z stack projection to obtain detailed images of PCs and other cells in the cerebellum. The scale bar is 100 μm. (C,D) Western blot analysis to evaluate the abundance of the ribosomal protein S6 in Control and PKO cerebellum. The study utilized 4 samples per group, and statistical comparisons were performed using unpaired student’s t-test. **p < 0.01. [file Image_3.TIF]

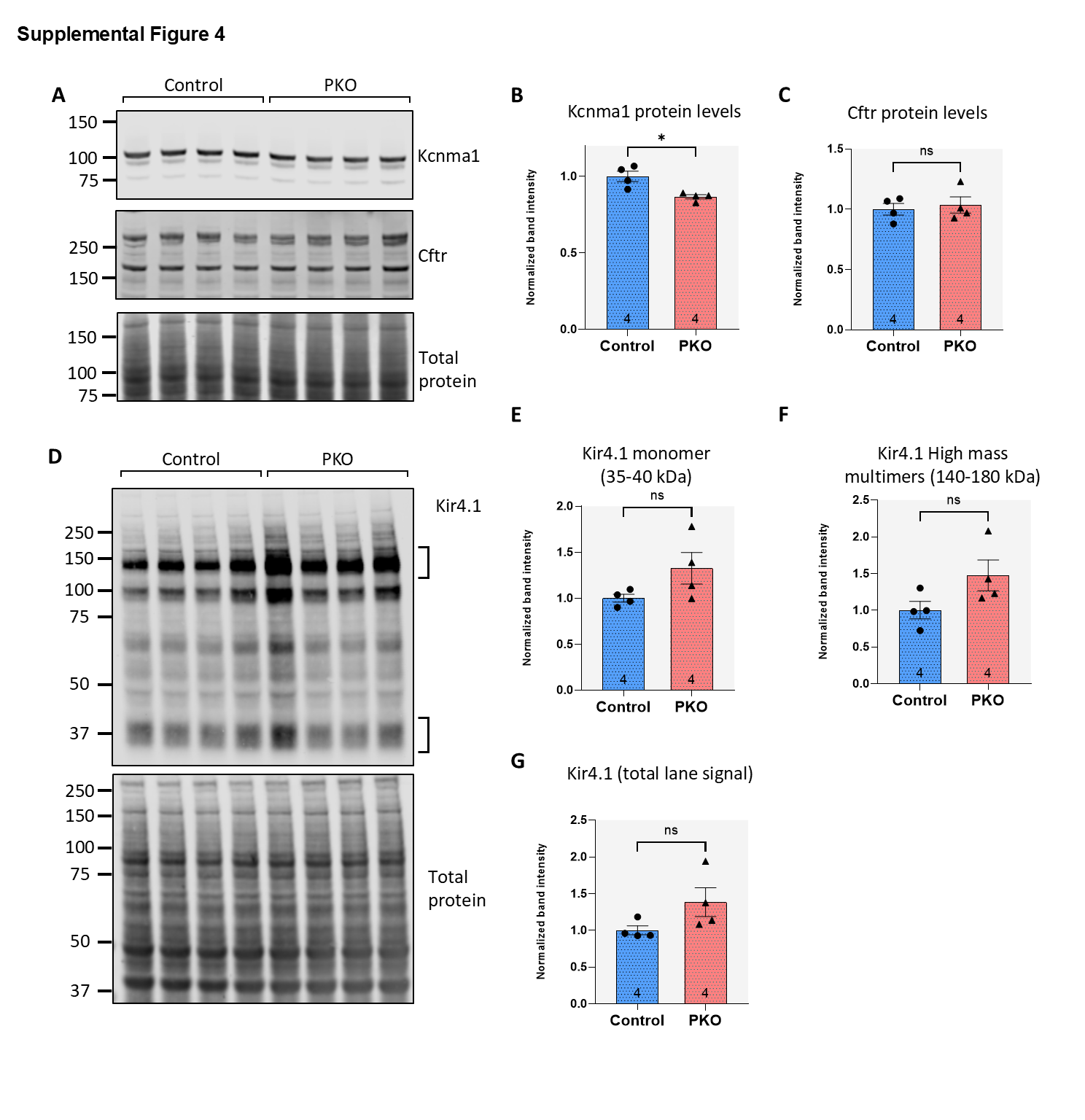

Supplement: SUPPLEMENTAL FIGURE 4 — Reduced protein levels of the big K+ channel subunit Kcnma1 in PKO cerebellum. (A–C) Western blot analysis in whole cerebellum lysates from Control and PKO samples (n = 4 biological replicates per group, 20 μg total protein loaded per lane) to assess the expression of ion channels Kcnma1 (Calcium-Activated Potassium Channel Subunit Alpha-1, also known as MaxiK or BK channel) and Cftr (Cystic Fibrosis Transmembrane Conductance Regulator). Band intensities corresponding to Kcnma1 were normalized to total protein loading and comparisons between Control and PKO samples were carried out using the unpaired Student’s t-test. *p < 0.05. (D) Western blot analysis of Kir4.1 in Control and PKO cerebellum. Brackets show expected Kir4.1 monomer ~ 35–40 kDa and higher-mass species ~ 140-180 kDa. (E–G) Normalized densitometry at the 35–40 kDa range (E), the 140-180 kDa range (F), and the total lane signal (30–250 kDa; including mid-MW bands at ~45–100 kDa) (G). Quantification was performed following normalization of each species to their respective total protein loading. Comparisons between Control and PKO samples were carried out using the unpaired Student’s t-test. The differences between Control and PKO groups are not significant (p = 0.11, 0.09, and 0.11 for panels E, F, and G, respectively). [file Image_4.TIF]

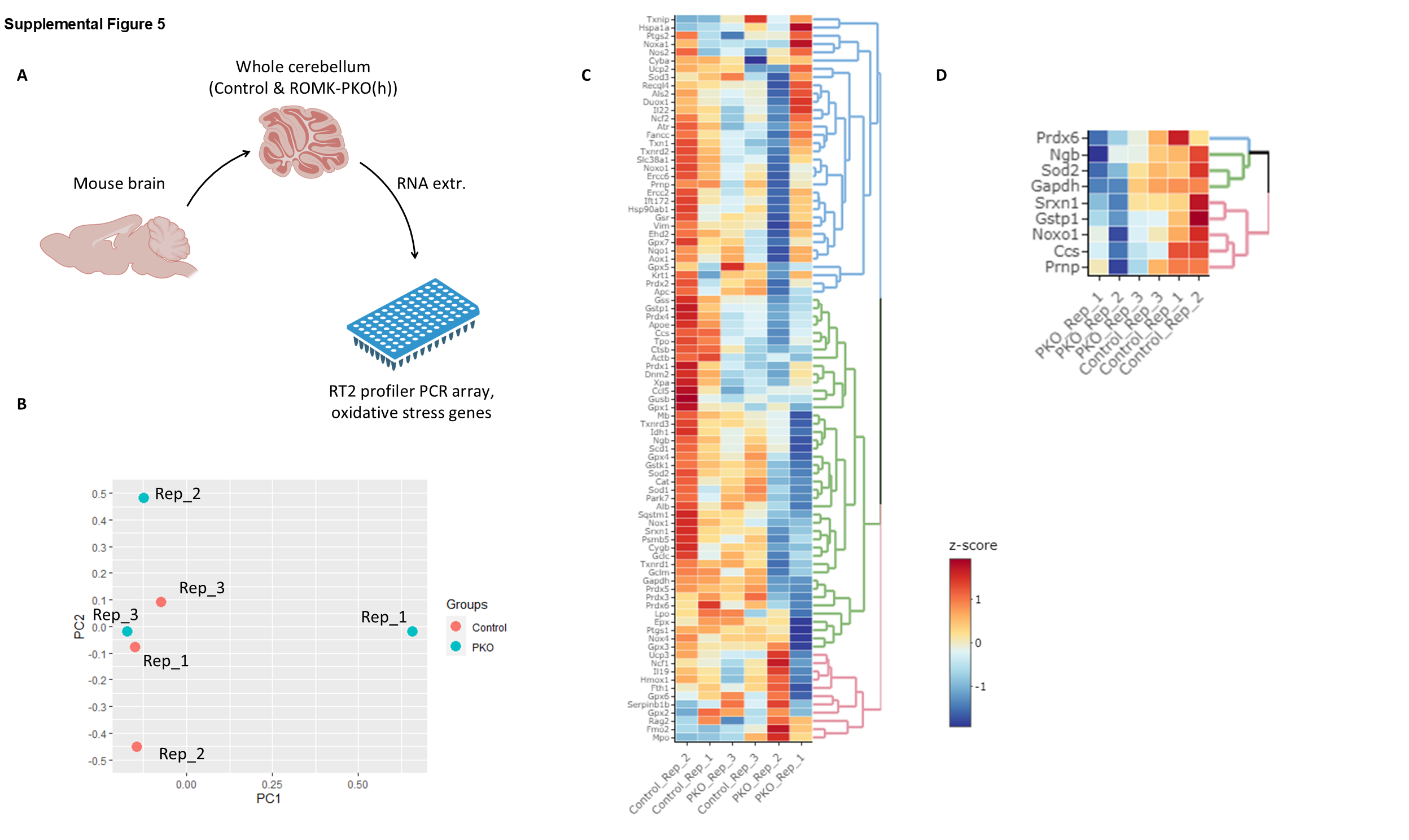

Supplement: SUPPLEMENTAL FIGURE 5 — Comparative expression analysis of oxidative stress genes in Control and PKO cerebellum. (A) Schematic of the experiment done to analyze the expression of oxidative stress genes in Control and PKO cerebellum. Total RNA was extracted using the Trizol reagent. 1280 ng of purified RNA was used to synthesize cDNA using the RT2 First Strand Kit. The resulting cDNA from each sample was combined with the RT2 SYBR Green qPCR mastermix and applied to 384-well plates pre-loaded with optimized primer pairs for Mouse Oxidative Stress genes, including negative controls and housekeeping genes (PAMM-065ZE). Real-time qPCR was performed on a CFX3984 system, and Ct values were obtained and normalized to that of B2M. Expression values for each gene per sample were log2 transformed and further analyzed statistically in R using the Linear Models for Microarray and RNA-seq data (limma) package and the Benjamini-Hochberg to control the false discovery rate during multiple hypothesis testing. (B) Principal Component Analysis (PCA) Plot was performed in R, using packages plotMDS (multidimensional scaling plot) and ggplot. Each data point corresponds to an individual sample, and 3 biological replicates per group (Rep_1–Rep_3) were included in the analysis. (C) Construction of the heatmap was performed in R using the package heatmaply. Gene expression values for each gene representing each row were z-scored by subtracting the row mean and dividing by the row standard deviation. The heatmap displays the relative expression levels of Mouse Oxidative Stress genes across Control and PKO cerebellum samples (89 genes total). The clustering was adjusted so that three major clusters of genes are defined as shown by the blue, green and magenta dendrograms. The intensity scale uses color-coded gradients, with warmer colors representing higher expression and cooler colors indicating lower expression levels. (D) Heatmap of selected oxidative stress genes from the PCR array that showed a trend for differ [file Image_5.TIF]

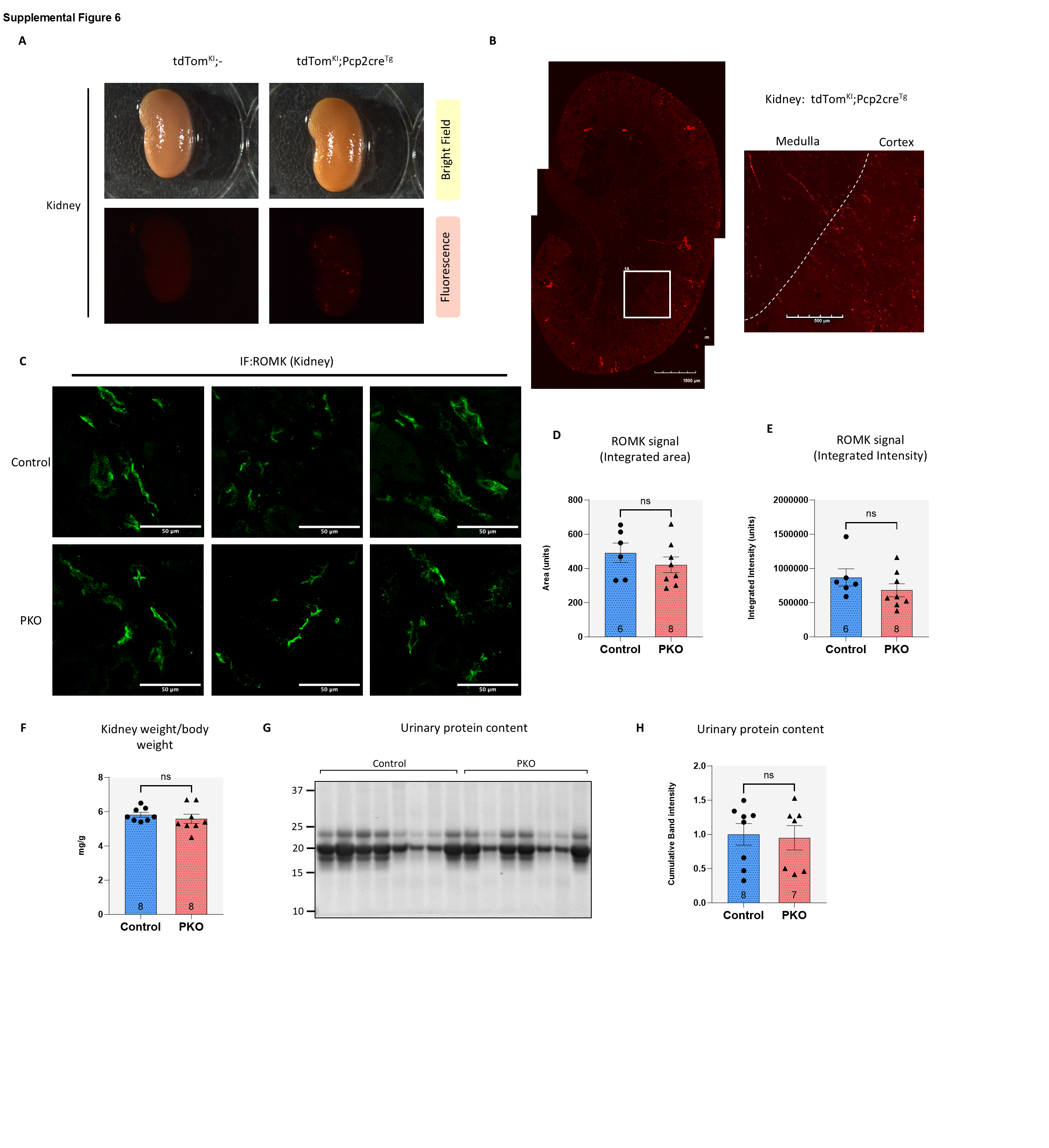

Supplement: SUPPLEMENTAL FIGURE 6 — Minimal tdTomato fluorescence in Pcp2cre kidney and normal ROMK expression and renal phenotype in PKO mice. (A) Macroscopic images of kidneys from tdTomatoKI;- and tdTomatoKI; Pcp2creTg mice shown in bright field and fluorescence using a fluorescent stereoscope. (B) Left: Fluorescent image of tdTomatoKI; Pcp2creTg kidney sectioned along the long axis to reveal the cortex and medulla, imaged with a confocal microscope (Olympus FV3000, Objective 4x). The scale bar is 1500 μm. Right: A zoomed region encompassing the cortex and medulla, shown at higher magnification. The scale bar in the right micrograph is 500 μm. Low but detectable tdTomato fluorescence above background can be observed in punctate formations in the cortex and elongated formations in the medulla, potentially indicating local Pcp2cre-expressing cell populations in the kidney. (C) Immunofluorescence (IF) staining of renal sections from Control and PKO mice showing polarized luminal expression pattern with tubular appearance, consistent with ROMK localization in renal epithelial cells. The imaging was performed using confocal microscopy with the 40× objective. (D,E) Confocal images were analyzed using Fiji/ImageJ to determine total ROMK expression in Control and PKO kidneys using area and integrated intensity measurements. Bar graphs represent aggregate data from multiple regions obtained from Control and PKO kidneys (n = 3 mice per genotype). No statistically significant differences were observed between groups (unpaired Student’s t-test). (F) Assessment of whole kidney mass normalized to body weight, as an index of kidney injury in Control and PKO mice (n=8 mice per group). No statistically significant difference in kidney mass was observed between the two groups (unpaired Student’s t-test). (G,H) Assessment of kidney function via total urinary protein analysis in Control and PKO mice. Urine was collected non-invasively, and samples were analyzed by reducing SDS-PAGE and western blot. Lane intensity was [file Image_6.TIF]
