# Supplementary material for: Characterizing molecular and behavioral changes arising from ROMK potassium channel deficiency in the cerebellum
Source: Front Behav Neurosci. 2026 Jan 26;19:1681149. doi: 10.3389/fnbeh.2025.1681149 (PMC12883771; doi:10.3389/fnbeh.2025.1681149)
Supplement: Supplementary file 7 [file Data_Sheet_1.PDF]

Supplemental Table 1. PCR primers used for genotyping

Locus	Primer name	Sequence (5´ to 3´)	Product (bp)
ROMK^{flox}	Intr_23542.For	GCCTGCTCCTGCTTTGCCATTTGCA	471 in ROMK^{WT} , 511 in ROMK^{flox}
	Gen_24102.Rev	GAGCCAAACAAGCCCTGCACAAGAGA	
Pcp2.cre^{Tg}	3395cre.For	GGCAATTTCGGCTATACGTAACAGGGT	473 in Pcp2.cre^{Tg}
	4468cre.Rev	GATGAGGTTCGCAAGAACCTGATGGA	
Rosa26^{WT}	RosaWT.For	GTGCAAGCACGTTTCCGACTTGA	1020 in Rosa26^{WT}
	RosaWT.Rev	GACGGGAGAGGTGATAGACACTGA	
tdTomato^{KI}	3908tdTom.For	GAGCAAGGGCGAGGAGGTCATCAAA	1457 in tdTom^{KI}
	5364tdTom.Rev	GGCCTTGTACGCGTTAAGTGCAACACAA	
ROMK^{WT}	Ex_27690.For	GGGGAGGTAGGGATAGGAGGATGTCT	462 in ROMK^{WT} and Het, absent in KO
	Ex_28151.Rev	GCACCATCTTGAGAGAGGAAGGAATAAAACC TCT	
ROMK$^{\Delta}$	Intr_30144.For	GGGCGGGAAACGCTCGGAAATTAA	675 in tissues with recombined ROMK$^{flox/flox}$ (ROMK$^{\Delta}$)
	Intr_36912.Rev	GCCTGAAGTGTGCTTGTGTCCAGA	

Supplemental Table 2. Primers used in real-time quantitative PCR

Target gene	Forward Sequence (5´ to 3´)	Reverse Sequence (5´ to 3´)	Accession No.
**Afg3l2**	CCA GAG TCC GAG ACT TAT TTG	TCC TTC CCA CAG CAT CAA TC	NM_027130.2
**Asic1**	CAG ATG GCT GAT GAA AAG CA	AAG TGG CAC GAG AGA AGC AT	NM_009597.2
**Atf4**	GCC ATC TCC CAG AAA GTT TAA TA	GGA ATG CTC TGG AGT GGA AGA C	NM_009716.3
**Atp5o**	CTA TGC AAC CGC CCT GTA CT	GTG CGC TTG ATG TAG GGA TT	NM_138597.2
**B2m**	CTG CTA CGT AAC ACA GTT CCA CCC	CAT GAT GCT TGA TCA CAT GTC TCG	NM_009735.3
**Bdnf**	GGA AAC GTG TCT CTC AGA ATG A	TCA TCC ACC TTG GCG ACT AC	NM_007540.4
**Ccdc51**	GGA TGC TAC AGG AAG AGA AG	CTT GGT CCT CTC AGC CCT TG	NM_025689.4
**Cox5b**	TAG ACT CCC ACC AAC GGA AG	ACT CCG CGA AGT AAC CTT GA	NM_009942.2
**Creb1**	AGA AGC AGC ACG GAA GAG AG	CAC TGC CAC TCT GTT CTC TAA A	NM_133828.2
**Dner**	TGC CAG GAC CAG TAC ATT GG	GCA AGT GAA ATT GCT CCC ATC C	NM_152915.1
**Drp1**	AGG AAC CAA CAA CAG GCA AC	ATG GCC TTT CTG TGG ACT TG	NM_001360008.2
**Eif4e**	AAC CGG AAA CCA CCC CTA C	CTC TGG GTT AGC AAC CTC CT	NM_007917.4
**Fis1**	AAG TAT GTG CGA GGG CTG TT	CCA TGC CTA CCA GTC CAT CT	NM_025562.3
**Gria1**	CTA GGC TGC CTG AAC CTT TG	GGG AAG ATT GAA TGG AAG CA	NM_001113325.2
**Grid2**	GTT GGT CTC GAA CCT GGG AC	CTG TGC GGA ATA CTT CAT CAT CT	NM_008167.3
**Homer3**	CAC GCA CTT ACC GTG TCC TA	GGG AGT GAC AGT GCT GTT GA	NM_001146153.1
**Hspd1**	CTG TTC TGG CAC GAT CTA TT	GCA TCC ACA GCC AAC ATC AC	NM_010477.4
**Hspe1**	TGT AAC CAA AGG TGG CAT TA	AAT CTC TCC ACT CTT TCC TTT C	NM_008303.4
**Kcna4**	ATC GTG GAG ACA GTG TGT ATT G	GCC CAG AGT GAT GAA GTA AGG	NM_021275.4
**Kcna5**	TGA GGA TGA GGA GGG AGA AG	CGC AAA CCC GAG ATG TTT ATG	NM_145983.2
**Kcnb1**	GGC TTG TAT CAC GAT CCT CTT AG	GCA CTT GCT GTG GTG TAG AT	NM_008420.4
**Kcnc1**	TCT GCA AAG CCT ACG GAT TC	AGG CTC AGC AAG GCT AAG G	NM_008421.3
**Kcnc3**	GAC GTA CCG CTC CAC GTT	CCG GGT CGT AGT CAA AGC	NM_008422.3
**Kcnd1**	GCC GCA GTA CCT CAG TAT CAT C	GAC AGA GGC AGT AGA GTT GGC A	NM_008423.2
**Kcnd2**	CGC CAC ATC CTC AAC TTC TA	CGG TCC TTG TAC TCC TCA TAA C	NM_019697.4
**Kcnd3**	GTG GCC ATC ATG CCC TAT TA	GAA GAT CCT GAA GAC ACG GAA G	NM_019931.2
**Kcnh2**	CTC ATG ACA CCA ACC ACA GG	GTT GTC CAT GGC AGA AAC CT	NM_013569.2
**Kcnip2**	TGA TAG ACT GAA CTG GGC TTT C	GTA GGT GTA CTT GCC CAT CAT	NM_145703.2
**Kcnj3**	AAA CTC ACT CTC ATG TTC CG	TCC AGT TCA AGT TGG TCA AG	NM_001355118.1
**Kcnj5**	GAG TTC GAA GTT GTG GTC ATA	GCA CCT CTG TAT CCA TGT AAG	NM_010605.6
**Kcnj6**	GAT GGG AAG TGC AAC GTT CA	AAG ATG TCC GTC AGG TAT CGG T	NM_010606.2
**Kcnj9**	TCG AGA GGG ACG ACT TCG AGA T	CCA GGT ACG AGC TTC GAG CTT	NM_008429.3
**Kcnj12**	AAG GGC CTA GAC CGT ATC TT	CTC AAA GTC GTC TGT CTC AAG G	NM_001267593.1
**Kcnj2**	GGT ACC TGG CAG ACA TCT TTA C	GAG CAG GGC TAT CAA CCA AA	NM_008425.4
**Kcnq1**	CAT CAC CCA TGT GTC ACA GC	CCG TGC TTG CTG GAA TTT CT	NM_008434.3
**Kcnq3**	TGC TGC TGG AAA CCT TTG C	CGA TAC AAA GGC TGG CGT	NM_152923.3
**Mfn1**	TGA AAG CTG GCT GTC TTG TG	AGA GCC GCT CAT TCA CCT TA	NM_024200.5
**Mfn2**	GAT TAC GGA GGA AGT GGA AAG G	AGA GAG GCG CCT GAT CTC TT	NM_133201.3
**Ndufb5**	TGG CAA GAG ACT GTT TGT CG	ACT CCC AGT GTT CAG GGA TG	NM_025316.2
**Opa1**	GGA AAG GAA CAC GAC GAC AT	CTT CCA AAG CAT TGT GCT GA	NM_133752.4
**Pcp2**	ACA GTT AAT TCC CTG CCT GG	CTC AAG GAG CTT GTG TCT GG	NM_001129804.2
**Ptpn11**	AGA GGG AAG AGC AAA TGT GTC A	CTG TGT TTC CTT GTC CGA CCT	NM_001109992.1
**Rgs6**	CTG ACA TTG TAC AGT GGC TTA T	GAG AAC ATG GTC TGA GAT TGG	NM_001413651.1
**Rictor**	ACA GTT GGA AAA GTG GCA CAA	GCG ACG AAC GTA GTT ATC ACC A	NM_030168.3

**Romk2**	CCA AGG TAC TGG GCA CCT TTA GC	TGC CGA GAA CGC CCA AAT ATG TG	NM_019659.3
**Rps6ka3**	ATG GAT GAA CCT ATG GGA GAG G	AAG CTG TCT AGC ATC AGA GCC	NM_148945.2
**Rptor**	TTT GTC TAC GAC TGT TCC AAT GC	GCT ACC TCT AGT TCC TGC TCC	NM_028898.3
**Scn1a**	CCC TAA GAG CCT TAT CAC GAT TT	TAA CAG GGC ATT CAC AAC CA	NM_001313997.1
**Slc1a6**	ACA ACA AGG CGA CAG GGC	TGC CGA TGA AAA CTG CAA TG	NM_009200.3
**Trap1**	GGC ACC CGC AAC ATC TAT TA	AAG AGC ACC TCA GTA TGT TTC	NM_026508.2
**Tsc1**	ATG GCC CAG TTA GCC AAC ATT	CAG AAT TGA GGG ACT CCT TGA AG	NM_001421615.1
**Tsc2**	AAA GAT TCC GGC TTG AAG GAG	GCA TTC ACC ACT CAG TTC TCT C	NM_011647.4
**Xbp**	AGA ACC AGG AGT TAA GGA CAC GC	CCA TGG GAA GAT GTT CTG GG	NM_001271730.1
**Xbp/s**	GGT CTG CTG AGT CCG CAG CAG G	GAA AGG GAG GCT GGT AAG GAA C	NM_001271730.1

Supplemental Table 3. Antibody list

Antibodies		
Rabbit polyclonal anti-Calbindin-D28k	Proteintech	Cat# 14479-1-AP
Mouse monoclonal anti-Calbindin	Proteintech	Cat# 66394-1-Ig
Rabbit polyclonal anti-Kcnj1 (ROMK)	Proteintech	Cat# 20953-1-AP
Rabbit monoclonal anti-S6 Ribosomal Protein (5G10)	Cell Signaling Technology	Cat# 2217
Rabbit monoclonal anti- Phospho-CREB (Ser133) (D1G6)	Cell Signaling Technology	Cat# 4276
Rabbit monoclonal anti- CREB (D76D11)	Cell Signaling Technology	Cat# 4820
Rabbit monoclonal anti- Phospho-p44/42 MAPK (Erk1/2) (Thr202/Tyr204) antibody	Cell Signaling Technology	Cat# 4370
Rabbit monoclonal anti- p44/42 MAPK (Erk1/2) (137F5)	Cell Signaling Technology	Cat# 4695
Rabbit polyclonal anti-KCNMA1 (K_{Ca}1.1) (1184-1200)	Alomone Labs	Cat# APC-107
Rabbit polyclonal anti-COX IV	Abcam	Cat# ab16056
Mouse monoclonal anti-UQCRQ	Abcam	Cat# ab110255
Mouse monoclonal anti-CFTR (CF3)	Invitrogen	Cat# MA1-935
Mouse monoclonal anti-Complex V, subunit alpha	Thermo	Cat# A-21350
Mouse monoclonal anti-Complex II, 30KDa subunit	Thermo	Cat# A-21345
Mouse monoclonal anti-GFAP (GA5)	Cell Signaling Technology	Cat# 3670
Rabbit anti-Ulk1 (R600)	Cell Signaling Technology	Cat# 4773
Rabbit monoclonal anti-p62/SQSTM1 (D1Q5S)	Cell Signaling Technology	Cat# 39749
Rabbit monoclonal anti-LC3B (D11)	Cell Signaling Technology	Cat# 39749
Rabbit polyclonal anti-TrKB	Abcam	Cat# ab18987
Rabbit monoclonal [EPR1292] anti-BDNF	Abcam	Cat# ab108319
Rabbit polyclonal anti-Kcnj10 (Kir4.1)	Proteintech	Cat# 12503-1-AP

Supplemental Table 4

**Supplemental Table 4**						
Antioxidant Genes PCR array, Limma statistics PKO vs. Control						
Gene_name	logFC	AveExpr	ß	*t*	P.Value	adj.P.Val
Ngb	-1.42	-6.21	-4.37	-2.82	0.03	0.53
Noxo1	-0.60	-7.34	-4.43	-2.23	0.06	0.53
Prnp	-0.43	0.12	-4.44	-2.21	0.06	0.53
Gapdh	-0.42	2.50	-4.45	-2.10	0.08	0.53
Sod2	-0.42	-0.01	-4.45	-2.10	0.08	0.53
Srxn1	-0.48	-2.37	-4.46	-2.06	0.08	0.53
Ccs	-0.42	-3.75	-4.46	-2.03	0.08	0.53
Gstp1	-0.46	-0.49	-4.46	-2.00	0.09	0.53
Prdx6	-0.36	-1.04	-4.48	-1.91	0.10	0.53
Cygb	-0.56	-2.59	-4.48	-1.89	0.10	0.53
Fmo2	0.82	-6.83	-4.48	1.88	0.10	0.53
Nox1	-0.51	-9.66	-4.48	-1.88	0.10	0.53
Gpx4	-0.34	0.79	-4.49	-1.84	0.11	0.53
Mb	-0.60	-7.13	-4.49	-1.82	0.11	0.53

Supplemental Table 4

Antioxidant Genes PCR array, Limma statistics PKO vs. Control

Gene_name	logFC	AveExpr	ß	*t*	P.Value	adj.P.Val
Tpo	-0.67	-9.17	-4.49	-1.82	0.11	0.53
Scd1	-0.36	-0.13	-4.49	-1.78	0.12	0.53
Prdx4	-0.34	-2.96	-4.50	-1.77	0.12	0.53
Psmb5	-0.32	-0.14	-4.50	-1.75	0.13	0.53
Ercc6	-0.35	-4.29	-4.50	-1.74	0.13	0.53
Ptgs1	-0.48	-5.39	-4.50	-1.73	0.13	0.53
Apoe	-0.32	2.25	-4.51	-1.69	0.14	0.53
Xpa	-0.37	-3.13	-4.51	-1.65	0.14	0.53
Sqstm1	-0.36	-0.77	-4.51	-1.64	0.15	0.53
Idh1	-0.33	-2.39	-4.52	-1.62	0.15	0.53
Prdx5	-0.29	0.36	-4.52	-1.60	0.16	0.53
Gclm	-0.26	-0.70	-4.52	-1.58	0.16	0.53
Serpinb1b	0.50	-3.48	-4.53	1.54	0.17	0.53
Gss	-0.28	-2.50	-4.53	-1.54	0.17	0.53
Alb	-1.28	-14.00	-4.53	-1.52	0.17	0.53

Supplemental Table 4

Antioxidant Genes PCR array, Limma statistics PKO vs. Control

Gene_name	logFC	AveExpr	ß	*t*	P.Value	adj.P.Val
Slc38a1	-0.30	-0.44	-4.54	-1.43	0.20	0.53
Cat	-0.26	-2.75	-4.54	-1.41	0.20	0.53
Dnm2	-0.29	-3.36	-4.55	-1.39	0.21	0.53
Prdx3	-0.24	-1.65	-4.55	-1.37	0.22	0.53
Txnrd3	-0.29	-6.16	-4.56	-1.32	0.23	0.53
Nox4	-0.76	-9.37	-4.56	-1.31	0.23	0.53
Prdx2	-0.36	-0.19	-4.56	-1.30	0.24	0.53
Ccl5	-0.33	-7.63	-4.56	-1.29	0.24	0.53
Hspa1a	0.89	-4.38	-4.57	1.26	0.25	0.53
Ctsb	-0.25	0.33	-4.57	-1.25	0.25	0.53
Gusb	-0.25	-4.64	-4.57	-1.25	0.25	0.53
Ercc2	-0.27	-4.16	-4.57	-1.23	0.26	0.53
Mpo	0.86	-11.02	-4.57	1.23	0.26	0.53
Ift172	-0.29	-3.04	-4.57	-1.23	0.26	0.53
Atr	-0.31	-3.23	-4.57	-1.21	0.27	0.53

Supplemental Table 4

Antioxidant Genes PCR array, Limma statistics PKO vs. Control

Gene_name	logFC	AveExpr	ß	*t*	P.Value	adj.P.Val
Sod1	-0.24	0.06	-4.57	-1.20	0.27	0.53
Park7	-0.22	-0.66	-4.58	-1.14	0.29	0.54
Gpx7	-0.40	-7.01	-4.58	-1.14	0.29	0.54
Gstk1	-0.19	-3.56	-4.58	-1.13	0.30	0.54
Prdx1	-0.29	-4.41	-4.59	-1.11	0.30	0.55
Hsp90ab1	-0.22	2.04	-4.59	-1.10	0.31	0.55
Txnrd2	-0.27	-5.05	-4.59	-1.07	0.32	0.56
Gclc	-0.22	-1.71	-4.59	-1.05	0.33	0.56
Vim	-0.42	-1.43	-4.60	-1.03	0.34	0.56
Ehd2	-0.26	-5.69	-4.60	-0.99	0.36	0.58
Nos2	0.83	-8.22	-4.61	0.94	0.38	0.59
Ncf2	-0.23	-5.05	-4.61	-0.94	0.38	0.59
Actb	-0.16	2.33	-4.61	-0.91	0.39	0.59
Hmox1	-0.20	-7.09	-4.61	-0.90	0.40	0.59
Epx	-0.42	-8.95	-4.61	-0.90	0.40	0.59

**Supplemental Table 4**						
Antioxidant Genes PCR array, Limma statistics PKO vs. Control						
Gene_name	logFC	AveExpr	ß	*t*	P.Value	adj.P.Val
Gsr	-0.20	-2.83	-4.61	-0.89	0.40	0.59
Txnrd1	-0.15	-2.81	-4.61	-0.89	0.41	0.59
Gpx3	-0.40	-3.47	-4.62	-0.87	0.42	0.59
Apc	-0.14	-1.73	-4.62	-0.81	0.45	0.63
Txn1	-0.28	-8.57	-4.63	-0.74	0.49	0.67
Cyba	0.49	-5.38	-4.63	0.73	0.49	0.67
Il19	-0.25	-12.74	-4.63	-0.71	0.50	0.67
Gpx1	-0.13	-0.53	-4.64	-0.68	0.52	0.68
Fancc	-0.22	-4.51	-4.64	-0.67	0.53	0.68
Il22	-0.29	-11.48	-4.64	-0.61	0.56	0.71
Fth1	-0.19	4.40	-4.64	-0.61	0.56	0.71
Noxa1	0.37	-14.37	-4.64	0.60	0.57	0.71
Als2	-0.24	-0.35	-4.65	-0.55	0.60	0.72
Gpx2	0.24	-9.13	-4.65	0.55	0.60	0.72
Recql4	-0.16	-6.62	-4.65	-0.47	0.65	0.77

Supplemental Table 4						
Antioxidant Genes PCR array, Limma statistics PKO vs. Control						
Gene_name	logFC	AveExpr	ß	*t*	P.Value	adj.P.Val
Txnip	0.12	-3.66	-4.65	0.46	0.66	0.77
Lpo	-0.14	-6.52	-4.66	-0.43	0.68	0.79
Duox1	-0.20	-4.69	-4.66	-0.37	0.72	0.82
Nqo1	-0.09	-4.35	-4.66	-0.36	0.73	0.82
Aox1	-0.07	-6.14	-4.66	-0.33	0.75	0.84
Ptgs2	-0.08	-8.32	-4.66	-0.30	0.78	0.85
Krt1	-0.10	-10.73	-4.67	-0.25	0.81	0.86
Ucp3	-0.08	-5.78	-4.67	-0.24	0.82	0.86
Rag2	0.12	-11.70	-4.67	0.23	0.82	0.86
Gpx6	0.20	-12.01	-4.67	0.23	0.82	0.86
Ucp2	0.05	-2.33	-4.67	0.18	0.86	0.89
Ncf1	-0.04	-6.52	-4.67	-0.13	0.90	0.93
Gpx5	-0.03	-16.01	-4.67	-0.09	0.93	0.94
Sod3	0.00	-3.88	-4.67	0.01	0.99	0.99
Note. n=3 per group						
